# Mindfulness‐Based Interventions for Depression, Anxiety, and Stress in Adults With Cancer: A Stratified Subgroup Meta‐Analysis

**DOI:** 10.1002/pon.70424

**Published:** 2026-03-12

**Authors:** Kenni Wojujutari Ajele, Erhabor Sunday Idemudia

**Affiliations:** ^1^ Faculty of Humanities North‐West University Mafikeng South Africa

## Abstract

**Background:**

Psychological distress, including depression, anxiety, and stress, is highly prevalent among adults with cancer. Mindfulness‐based interventions (MBIs), such as Mindfulness‐Based Stress Reduction (MBSR) and Mindfulness‐Based Cognitive Therapy (MBCT), are increasingly used to address these symptoms. However, no prior review has comprehensively stratified MBI effects across intervention types, symptom domains, cancer populations, and geographic regions.

**Aims:**

To evaluate the effectiveness of MBIs, including standard and adapted formats, on depression, anxiety, and stress in adults with cancer.

**Methods:**

A systematic review and meta‐analysis of randomized controlled trials (RCTs) was conducted in accordance with PRISMA guidelines. Six databases were searched from January 2010 to July 2025. Eligible studies included RCTs comparing MBIs to control conditions in adults with cancer, reporting validated outcomes for depression, anxiety, or stress. Random‐effects meta‐analyses were performed using Hedges’ *g*, with subgroup analyses by intervention type, duration, geographic region, and cancer type.

**Results:**

Across 84 effect sizes from 45 RCTs (*N* = 7395), mindfulness‐based interventions were evaluated. MBIs significantly reduced depression (*g* = −0.92), anxiety (*g* = −1.06), and stress (*g* = −1.50). Modified MBIs MBIs demonstrated the largest effects (*g* = −1.57), followed by MBSR (*g* = −0.72) and MBCT (*g* = −0.68). The strongest effects were observed in breast cancer populations (*g* = −1.48) and in studies conducted in North America (*g* = −1.21) and Asia (*g* = −1.07).

**Conclusions:**

Mindfulness‐based interventions (MBIs) were associated with reduced depression, anxiety, and stress in adults with cancer, though heterogeneity was high and evidence was largely from breast cancer trials. MBIs appear scalable, particularly for women with breast cancer, but broader conclusions remain limited. Findings support their inclusion in tailored psychosocial care, with a need for more diverse and rigorously controlled research.

## 1 Introduction

Cancer remains a leading global cause of disability and mortality, with nearly 10 million deaths in 2020 [1, 2]. Psychological distress is common following diagnosis and treatment, with depression, anxiety, and stress affecting up to 60% of individuals [3, 4, 5, 6]. These symptoms are linked to poor treatment adherence, lower quality of life, and higher mortality [5, 6]. To inform supportive care, psychosocial interventions must be evaluated. We assessed the effectiveness of mindfulness‐based interventions (MBIs), including mindfulness‐based stress reduction (MBSR), mindfulness‐based cognitive therapy (MBCT), and adapted formats, on psychological outcomes in adults with cancer.

Mental health burden varies by cancer type. Researchers frequently study depression, anxiety, and stress in breast, genitourinary, hematologic, ovarian, and head and neck cancers [7, 8, 9]. Distress levels depend on disease site, stage, recurrence risk, and treatment burden [10, 11]. Breast cancer survivors may experience anxiety or depression, while advanced illness patients may have existential distress [11]. Previous meta‐analyses have aggregated randomized clinical trial (RCT) data on MBIs’ customized benefits for cancer types or mental health outcomes, but they have not compared or stratified evidence across intervention types and psychological domains.

Although pharmacologic treatments exist, their use may be limited by side effects, stigma, cost, or access [12, 13, 14]. MBIs offer a nonpharmacologic approach to improve psychological well‐being. Structured mindfulness programmes like MBSR and MBCT promote emotion regulation through present‐moment awareness and nonjudgmental attention [15, 16, 17, 18, 19]. Oncology‐adapted programs, including brief or digital formats, have been developed to improve accessibility [20, 21].

Previous reviews support the general benefits of MBIs but rarely compare outcomes across different intervention types, symptom domains, program lengths, or regions [22, 23, 24, 25]. We extend existing evidence by comparing the effectiveness of MBSR, MBCT, and adapted MBIs on depression, anxiety, and stress, with stratified analyses by intervention type, duration, and geographic location.

## 2 Methods

### 2.1 Search Strategy

We adhered to the PRISMA (Preferred Reporting Items for Systematic Reviews and Meta‐analyses) guidelines [26]. We searched Web of Science, Scopus, ScienceDirect, and EBSCOhost (including MEDLINE, CINAHL, and APA PsycInfo), covering the period from January 2010 through July 2025. Search terms targeted mindfulness‐based interventions, cancer populations, and psychological outcomes. Core terms included “mindfulness”, “MBSR,” “MBCT,” “cancer,” “oncology,” “depression,” “anxiety,” “stress”, and “RCT” with filters applied to identify randomized controlled trials. Complete search strategies for each database are detailed in Supporting Information S1: eTable 1 in the supplement. The reference lists of included studies and prior systematic reviews were also screened to capture additional eligible trials.

### 2.2 Eligibility Criteria

We included randomized controlled trials (RCTs) that enrolled adults aged 18 years or older with a medically confirmed diagnosis of cancer, either during active treatment or in survivorship. The focus on adults ensured greater consistency in psychological treatment response because pediatric populations differ developmentally and typically require distinct therapeutic approaches. Trials that focused exclusively on palliative or end‐of‐life care were excluded due to their unique clinical and psychological needs.

Eligible studies evaluated a mindfulness‐based intervention (MBI), including standard programs such as Mindfulness‐Based Stress Reduction (MBSR) and Mindfulness‐Based Cognitive Therapy (MBCT), as well as adapted or modified formats developed for oncology populations. MBIs were defined as structured programs that provided mindfulness training through practices such as meditation, body scanning, or mindful movement, and were aligned with established treatment frameworks. This ensured conceptual clarity and consistency across interventions.

Studies were required to include a comparison group such as a waitlist, treatment as usual, attention control, or another active control condition. Eligible studies also had to report psychological outcomes related to depression, anxiety, or stress using validated instruments with established reliability. Commonly accepted measures included the Hospital Anxiety and Depression Scale (HADS), Patient Health Questionnaire (PHQ), Depression Anxiety and Stress Scales (DASS), Beck Depression Inventory (BDI), Generalized Anxiety Disorder scale (GAD‐7), and Perceived Stress Scale (PSS).

To allow for standardized effect size calculation, studies were required to report pre‐ and post‐treatment means and standard deviations for both the intervention and control groups. This enabled the use of change scores that reflect differences from baseline to post‐treatment. Studies were excluded if they did not include a comparator group, used non‐randomized designs, lacked extractable pre‐ and post‐treatment data, or provided insufficient statistical details. Abstract‐only publications, protocols without outcome data, and duplicate reports without unique results were also excluded. Trials involving mixed medical populations were excluded unless separate cancer‐specific results were available.

### 2.3 Study Selection and Data Extraction

Two reviewers (KWA and ESI) independently screened all titles, abstracts, and full‐text articles to determine eligibility. We resolved disagreements through discussion and, if necessary, adjudication by a third reviewer. A standard data extraction form was used to collect information on the study”s design, country, sample size, cancer type, participant demographics (including age and gender), intervention characteristics (type, duration, and delivery mode), comparator groups, psychological outcomes, and outcome measures. Studies reporting more than one psychological domain or subgroup were coded separately, yielding a total of 85 effect sizes across 46 randomized controlled trials.

### 2.4 Risk of Bias Assessment

The risk of bias across included studies was assessed using the Cochrane Risk of Bias Tool 2.0 (RoB 2) [27], which evaluates five domains: (1) bias from the randomization process, (2) deviations from intended interventions, (3) missing outcome data, (4) outcome measurement, and (5) selection of reported results. As illustrated in Supporting Information S1: eFigure 1, most studies were rated as having low risk across domains (green), while several studies showed either “some concerns” (yellow) or high risk (red), especially in Domains 1 and 4. These findings suggest generally acceptable study quality, though improvements in randomization and outcome assessment procedures are needed.

### 2.5 Statistical Analysis

Continuous outcomes were synthesized as standardized mean differences (Hedges’ *g*), computed using change scores. Specifically, we calculated the difference from baseline to post‐treatment in the intervention group and subtracted the corresponding change in the control group. Effect sizes were computed using the escalc() function from the metafor package in R, with the syntax “escalc(measure = “SMD”, m1i = post_mean, m2i = baseline_mean, ..) to capture pre‐post differences”. A negative g indicates a beneficial effect of the mindfulness‐based intervention (MBI). Random‐effects models were used with τ^2^ estimated via restricted maximum likelihood (REML) and Hartung–Knapp confidence intervals. We report 95% confidence intervals (CIs), Cochran’s Q, τ^2^, and *I* ^2^. Subgroup meta‐analyses (via byvar) examined mental health condition (depression, anxiety, stress, or both), MBI type (MBSR, MBCT, MBCR, or adapted/modified), continent (Asia, Europe, North America, or Oceania; unmatched entries were grouped as “Other”), dosage based on total hours (< 16 vs. ≥ 16) and weeks (< 8 vs. ≥ 8), and cancer population (breast, genitourinary, gynecologic, hematologic, mixed cancer survivors, or other). When intervention duration was given as a range (e.g., 8–12 weeks), the midpoint was used. Dropout rate was analyzed as < 10% versus ≥ 10%; when only counts were reported, percentages were calculated based on the total randomized sample (intervention + control). Values ≤ 1 were treated as proportions and multiplied by 100. Ranges were averaged, and expressions such as “≥ 94%” were coded as 94%. The unit of analysis was the study‐outcome comparison. Trials contributing multiple outcomes were analyzed within the appropriate subgroup. Rows missing essential numeric fields (means, standard deviations, or sample sizes) were excluded; missing duration values were excluded only from duration‐based subgrouping. Small‐study effects were planned to be assessed using funnel plots and Egger’s test when k ≥ 10, with trim‐and‐fill used for exploratory purposes [28]. All analyses were conducted in R (RStudio 2025.05.1 + 501) using the metafor, meta, and readxl packages. Forest plots were generated using meta::forest() (see Supplement for code).

## 3 Results

### 3.1 PRISMA Flow Diagram

**FIGURE 1:**
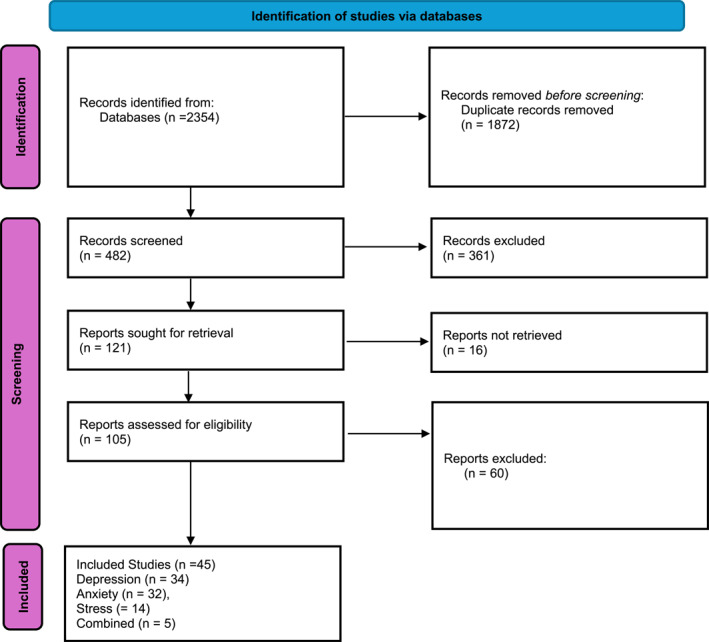
PRISMA flow diagram of study selection for the meta‐analysis.

A total of 2354 records were identified through database searches. After removing 1872 duplicates, 482 unique records were screened based on title and abstract. Of these, 361 were excluded for not meeting inclusion criteria. Full texts of 121 reports were sought for retrieval; 16 could not be accessed. Of the 105 full‐text articles reviewed, 60 were excluded due to ineligible population, outcomes, or study design. Ultimately, 45 randomized controlled trials were included in the meta‐analysis (Figure 1).

### 3.2 Characteristics of the Included Studies

In our meta‐analysis, we included 45 randomized controlled trials, contributing 85 effect sizes across depression (*k* = 34), anxiety (*k* = 32), stress (*k* = 14), and combined mental health outcomes (*k* = 5) (Figure 1). Several studies contributed multiple outcomes, either reported independently [29, 30, 31] or as composite scores [32, 44]. Effect sizes were derived from both distinct psychological domains and independent subgroups. Across the 45 included trials, most participants were female (approximately 87%), with breast cancer being the most common diagnosis (present in 68% of studies) [29, 30, 31]. Most trials focused on survivors or individuals’ post‐treatment, with limited representation of participants undergoing active treatment or with advanced‐stage disease [14, 33]. Mean participant age ranged from 30 to 70 years, with most samples composed of middle‐aged adults. A smaller number of trials included individuals with prostate, hematologic, gynecologic, lung, colorectal, or other cancers [34, 35].

Interventions included MBSR, MBCT, and adapted MBIs. The duration ranged from 1 to 20 weeks (median 6–8 weeks), with the total program contact time ranging from 2.3 to 72 h. MBIs were delivered in group, individual, or digital formats. [36, 44] Psychological outcomes were assessed using validated instruments, including the Hospital Anxiety and Depression Scale (HADS), Perceived Stress Scale (PSS), Center for Epidemiological Studies Depression Scale (CES‐D), Generalised Anxiety Disorder‐7 (GAD‐7), and Patient Health Questionnaire‐9 (PHQ‐9) (Supporting Information S1: eTable 2). All studies reported pre/post data for both intervention and control groups [31, 37]. Studies were conducted across North America, Asia, Europe, and Oceania. Dropout rates vary depending on the delivery method and program length (Supporting Information S1: eTable 2).

### 3.3 MBIs on Mental Health Outcomes

**FIGURE 2:**
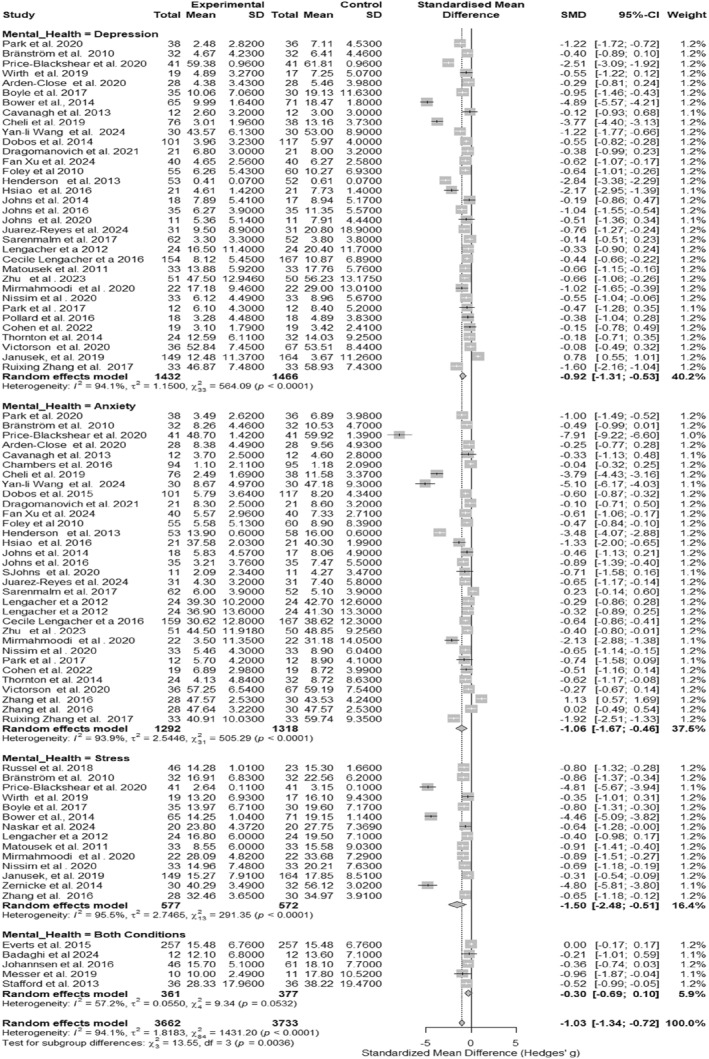
Forest plot of overall effects of mindfulness‐based interventions on depression, anxiety, stress, and combined outcomes.

A meta‐analysis of 45 randomized clinical trials involving 7395 adults with cancer (3662 in mindfulness‐based intervention [MBI] groups and 3733 in control groups) found that MBIs were associated with a large overall reduction in psychological symptoms, including depression, anxiety, and stress (Hedges’ *g* = −1.03; 95% CI: −1.33 to −0.74; Table 1, Figure 2). The 95% prediction interval ranged from −3.69 to 1.63, suggesting substantial heterogeneity and cautioning that future studies may show smaller or even null effects. When outcomes were analyzed separately, MBIs showed clinically meaningful improvements for depression (*g* = −0.92; 95% CI: −1.31 to −0.53), anxiety (*g* = −1.06; 95% CI: −1.67 to −0.46), and stress (*g* = −1.50; 95% CI: −2.48 to −0.51; Table 1). In contrast, trials that measured all three symptom domains jointly showed a smaller effect size (*g* = −0.30; 95% CI: −0.69 to 0.10). All three MBI formats were associated with reductions in psychological symptoms, with the strongest effects seen for adapted or modified MBIs (e.g., brief, digital, or oncology‐specific programs) (*g* = −1.57; 95% CI: −2.22 to −0.92), followed by MBSR (*g* = −0.72; 95% CI: −1.08 to −0.35) and MBCT (*g* = −0.68; 95% CI: −1.20 to −0.17) (Table 1). These findings support the potential value of MBIs for mental health in oncology, while highlighting the variability in effects across study designs and populations.

**TABLE 1:** Main outcomes of mindfulness‐based interventions for depression, anxiety, and stress in adults with cancer.

Subgroup	No. of studies (k)	Hedges’ *g* (95% CI)	*I* ^2^(%)	Between‐group *p*‐value
Overall effect	85	−1.03 (−1.34 to −0.72)	94.1	—
Mental health outcome				0.0036
Depression	34	−0.92 (−1.31 to −0.53)	94.1	
Anxiety	32	−1.06 (−1.67 to −0.46)	93.9	
Stress	14	−1.50 (−2.48 to −0.51)	95.5	
Both conditions	5	−0.30 (−0.69 to 0.10)	57.2	
MBI type				0.0453
MBSR	36	−0.72 (−1.08 to −0.35)	92.4	
MBCT	17	−0.68 (−1.20 to −0.17)	82.2	
Adapted/Modified MBIs	32	−1.57 (−2.22 to −0.92)	96.1	
Continent				0.0044
Asia	21	−1.07 (−1.58 to −0.56)	88.8	
Europe	13	−0.77 (−1.58 to 0.04)	95.3	
North America	43	−1.21 (−1.73 to −0.69)	95.5	
Oceania	8	−0.42 (−0.64 to −0.20)	36.8	
MBI duration (total hours)				0.1815
Less than 16 h	41	−1.25 (−1.80 to −0.71)	95.1	
16 h or more	44	−0.83 (−1.16 to −0.50)	93	
MBI duration (Weeks)				0.4677
Fewer than 8 weeks	28	−0.89 (−1.33 to −0.45)	91.9	
8 weeks or more	57	−1.11 (−1.52 to −0.69)	94.8	
Dropout rate				0.4749
Less than 10%	40	−1.21 (−1.72 to −0.71)	94.9	
10% or more	24	−0.94 (−1.54 to −0.33)	93.7	

*Note:* Both Conditions refers to composite or combined measures (anxiety depression and stress). A higher *I* ^2^ means more variation (or heterogeneity) between studies. Abbreviations: *g*, Hedges’ *g*; CI, confidence interval; MBI, mindfulness‐based intervention; MBSR, Mindfulness‐Based Stress Reduction; MBCT, Mindfulness‐Based Cognitive Therapy.

### 3.4 Geographic Region

Benefits of MBIs were consistent across continents. The largest effects were observed in studies conducted in North America (*g* = −1.21; 95% CI, −1.73 to −0.69) and Asia (*g* = −1.07; 95% CI, −1.58 to −0.56), with smaller but still meaningful effects in Europe (*g* = −0.77; 95% CI, −1.58 to 0.04) and Oceania (*g* = −0.42; 95% CI, −0.64 to −0.20) (Table 1).

### 3.5 MBI Duration and Dropout Rate

MBIs lasting fewer than 16 total hours were associated with larger effects (*g* = −1.25; 95% CI, −1.80 to −0.71) compared with those lasting 16 h or longer (*g* = −0.83; 95% CI, −1.16 to −0.50). Similarly, programs of 8 weeks or longer yielded stronger effects (*g* = −1.11; 95% CI, −1.52 to −0.69) than shorter ones (*g* = −0.89; 95% CI, −1.33 to −0.45) (Table 1). Trials with lower dropout rates (< 10%) showed stronger effects (*g* = −1.21; 95% CI, −1.72 to −0.71) than those with higher dropout rates (≥ 10%) (*g* = −0.94; 95% CI, −1.54 to −0.33), suggesting better retention may enhance clinical impact (Table 1).

### 3.6 Mental Health Outcome and Continent

MBIs were associated with substantial reductions in depression, anxiety, and stress symptoms in adults with cancer. The overall effect was clinically large (*g* = −1.03; 95% CI, −1.34 to −0.72), indicating meaningful benefits compared with control conditions (Table 2A). Effectiveness varied by intervention type and symptom domain. Modified MBIs or modified MBIs yielded the largest effects (*g* = −1.57; 95% CI, −2.22 to −0.92). These interventions were especially effective for anxiety (*g* = −1.75; 95% CI, −3.44 to −0.06), stress (*g* = −1.96; 95% CI, −4.12 to 0.20), and depression (*g* = −1.45; 95% CI, −2.27 to −0.63). MBSR showed moderate effects across outcomes, including depression (*g* = −0.60; 95% CI, −1.03 to −0.17), anxiety (*g* = −0.88; 95% CI, −1.72 to −0.04), and stress (*g* = −0.61; 95% CI, −0.91 to −0.32). MBCT was also beneficial overall (*g* = −0.68; 95% CI, −1.20 to −0.17), with symptom‐specific effects observed for depression (*g* = −0.48; 95% CI, −0.76 to −0.20) and anxiety (*g* = −0.50; 95% CI, −0.81 to −0.19). The effect on stress was highly variable (*g* = −2.72; 95% CI, −28.86 to 23.42) (Table 2A).

**TABLE 2:** Effects of mindfulness‐based intervention types by mental health outcome and continent.

	Outcome	No. of studies (k)	Hedges’ *g* (95% CI)	*I* ^2^ (%)	Between‐group *p*‐value
A. By mental health					
Adapted/Modified MBIs					0.173
	Overall	32	−1.57 (−2.22 to −0.92)	96.10	
	Depression	14	−1.45 (−2.27 to −0.63)	94.80	
	Anxiety	10	−1.75 (−3.44 to −0.06)	95.80	
	Stress	6	−1.96 (−4.12 to 0.20)	97.10	
	Both conditions	2	−0.37 (−6.29 to 5.55)	75.40	
MBSR					0.7986
	Overall	36	−0.72 (−1.08 to −0.35)	92.40	
	Depression	15	−0.60 (−1.03 to −0.17)	93.10	
	Anxiety	15	−0.88 (−1.72 to −0.04)	94.20	
	Stress	6	−0.61 (−0.91 to −0.32)	43.90	
MBCT					0.5811
	Overall	17	−0.68 (−1.20 to −0.17)	82.20	
	Depression	5	−0.48 (−0.76 to −0.20)	0.00	
	Anxiety	7	−0.50 (−0.81 to −0.19)	57.30	
	Stress	2	−2.72 (−28.86 to 23.42)	98.10	
	Both conditions	3	−0.40 (−0.71 to −0.08)	0.00	
B. By continent					
Adapted/Modified MBIs					0.3158
	Overall	32	−1.57 (−2.22 to −0.92)	96.1	
	Asia	8	−1.23 (−1.73 to −0.73)	76	
	Europe	5	−1.61 (−4.06 to 0.84)	98.3	
	North America	18	−1.76 (−2.84 to −0.67)	96.3	
	Oceania	1	−0.80 (−1.32 to −0.28)	—	
MBSR					0.3241
	Overall	36	−0.72 (−1.08 to −0.35)	92.4	
	Asia	10	−1.05 (−2.20 to 0.09)	93.3	
	Europe	4	−0.28 (−0.89 to 0.33)	81	
	North America	21	−0.68 (−1.09 to −0.27)	93.4	
MBCT	Oceania	1	−0.38 (−1.04 to 0.28)	—	0.0052
	Overall	17	−0.68 (−1.20 to −0.17)	82.2	
	Asia	3	−0.84 (−1.50 to −0.18)	0	
	Europe	4	−0.30 (−0.46 to −0.14)	0	
	North America	4	−1.63 (−4.92 to 1.65)	95.1	
	Oceania	6	−0.37 (−0.64 to −0.11)	40.2	

*Note:* Both Conditions refers to composite or combined measures (anxiety depression and stress). A higher *I* ^2^ means more variation (or heterogeneity) between studies.

Abbreviations: *g*, Hedges’ *g*; CI, confidence interval; MBI, mindfulness‐based intervention; MBCT, Mindfulness‐Based Cognitive Therapy; MBSR, Mindfulness‐Based Stress Reduction.

Modified MBIs or modified MBIs showed the strongest effects in North America (*g* = −1.76; 95% CI, −2.84 to −0.67) and Asia (*g* = −1.23; 95% CI, −1.73 to −0.73), with more variability in Europe (*g* = −1.61; 95% CI, −4.06 to 0.84) and Oceania (*g* = −0.80; 95% CI, −1.32 to −0.28). MBSR demonstrated consistent effects across North America (*g* = −0.68; 95% CI, −1.09 to −0.27) and more modest results in Europe (*g* = −0.28; 95% CI, −0.89 to 0.33). Asian studies showed larger but less precise effects (*g* = −1.05; 95% CI, −2.20 to 0.09). MBCT was most effective in Asia (*g* = −0.84; 95% CI, −1.50 to −0.18) (Table 2B). Smaller but clinically meaningful effects were found in Europe (*g* = −0.30; 95% CI, −0.46 to −0.14) and Oceania (*g* = −0.37; 95% CI, −0.64 to −0.11). Effects were large but highly variable in North America (*g* = −1.63; 95% CI, −4.92 to 1.65) (Table 2B).

### 3.7 MBIs Across Cancer Types and Mental Health Outcomes

Stratified subgroup analyses revealed variation in effect sizes across cancer types, intervention formats, and mental health outcomes (Supporting Information S1: eTable 3). The strongest effects were observed in breast cancer populations (Hedges’ *g* = −1.48; 95% CI: −2.03 to −0.93) and hematologic cancers (*g* = −1.75; 95% CI: −3.78 to 0.28). Smaller effects were reported for gynecologic cancers (*g* = −0.29; 95% CI: −0.51 to −0.07), with minimal change among genitourinary cancers (*g* = −0.08; 95% CI: −0.34 to 0.19) (Table 3). Across intervention types, adapted or modified MBIs showed the largest reductions in psychological symptoms (*g* = −1.57; 95% CI: −2.22 to −0.92), particularly among participants with breast cancer (*g* = −2.43; 95% CI: −3.51 to −1.35). MBSR yielded moderate effects (*g* = −0.72; 95% CI: −1.08 to −0.35), and MBCT showed similar results (*g* = −0.68; 95% CI: −1.20 to −0.17), both with stronger effects in breast cancer samples (Table 3). When stratified by psychological outcome, MBIs were associated with reduced symptoms of depression (*g* = −0.92; 95% CI: −1.31 to −0.53), anxiety (*g* = −1.06; 95% CI: −1.67 to −0.46), and stress (*g* = −1.50; 95% CI: −2.48 to −0.51) (Supporting Information S1: eTable 3). Breast cancer groups showed the largest improvements across all outcomes. Given the heterogeneity in populations and intervention characteristics, these subgroups' findings should be interpreted with caution.

**TABLE 3:** Effects of mindfulness‐based interventions by cancer population group.

Subgroup	No. of studies (k)	Hedges’ *g* (95% CI)	*I* ^2^ (%)	Between‐group *p*‐value
Overall (all MBIs)	85	−1.03 [–1.34, −0.72]	94.1	< 0.0001
Breast cancer	40	−1.48 [–2.03, −0.93]	95.8	
Genitourinary cancer	3	−0.08 [–0.34, 0.19]	0	
Gynecologic cancer	4	−0.29 [–0.51, −0.07]	0	
Hematologic cancer	2	−1.75 [–3.78, 0.28]	0	
Mixed cancer survivors	8	−0.44 [–0.67, −0.21]	71.9	
Other cancer types	28	−0.70 [–1.15, −0.24]	89.9	
Adapted/Modified MBIs only	32	−1.57 [–2.22, −0.92]	96.1	< 0.0001
Breast cancer	17	−2.43 [–3.51, −1.35]	96.7	
Gynecologic cancer	2	−0.27 [–0.52, −0.01]	0	
Hematologic cancer	2	−1.75 [–3.78, 0.28]	0	
Mixed cancer survivors	3	−0.18 [–0.87, 0.51]	38	
Other cancer types	8	−0.49 [–0.74, −0.24]	0	
MBSR only	36	−0.72 [–1.08, −0.35]	92.4	0.571
Breast cancer	18	−0.84 [–1.37, −0.32]	93.2	
Mixed cancer survivors	5	−0.57 [–0.73, −0.42]	0	
Other cancer types	13	−0.61 [–1.42, 0.19]	92.7	
Subgroup *p*‐value				
MBCT only	17	−0.68 [–1.20, −0.17]	82.2	0.0007
Breast cancer	5	−0.60 [–0.95, −0.25]	11.6	
Genitourinary cancer	3	−0.08 [–0.34, 0.19]	0	
Gynecologic cancer	2	−0.33 [–2.62, 1.97]	0	
Other cancer types	7	−1.10 [–2.56, 0.35]	91.1	

*Note:* Both Conditions refers to composite or combined measures (anxiety depression and stress). A higher *I* ^2^ means more variation (or heterogeneity) between studies. Abbreviations: *g*, Hedges’ *g*; CI, confidence interval; MBCT, Mindfulness‐Based Cognitive Therapy; MBI, mindfulness‐based intervention; MBSR, Mindfulness‐Based Stress Reduction.

### 3.8 Clinical Interpretation of Effect Sizes

To improve interpretability, pooled standardized mean differences (SMDs) were translated into estimated raw score changes using typical baseline standard deviations. Results suggest meaningful symptom reductions across measures (Supporting Information S1: eFigure 3). The largest estimated changes were seen on STAI and PROMIS‐A (−10.6 points), PSS (−9.75), and CES‐D and PROMIS‐D (both −9.2). Depression scores declined by −3.9 to −9.2 points across scales; anxiety and stress showed comparable patterns. These estimates suggest that MBIs yield clinically relevant improvements in psychological symptoms, though estimates are approximate.

### 3.9 Subgroup Meta‐Regression by Mental Health Outcome

We conducted a multilevel RVE meta‐regression to examine whether intervention effects differed by mental health outcome type (i.e., anxiety, depression, stress, or both conditions). The model included 85 effect sizes from 49 clusters. A significant overall moderation effect was observed (*F*(3, 45) = 3.41, *p* = 0.025), explaining some between‐study variability (Supporting Information S1: eTable 4). Compared to anxiety (reference group; *g* = −0.93, 95% CI [–1.36, −0.49]), studies targeting both depression and anxiety yielded significantly larger effects (*b* = 0.53, 95% CI [0.01, 1.06], *p* = 0.048) (Supporting Information S1: eTable 4). Effect sizes for depression‐only studies were slightly larger (*b* = 0.15, 95% CI [–0.04, 0.35]), while stress outcomes had smaller, non‐significant effects (*b* = −0.36, 95% CI [–0.86, 0.15]) (Supporting Information S1: eTable 4). The pooled Hedges’ *g* across all studies was −0.88 (95% CI [–1.21, −0.54]), with high residual heterogeneity (*I* ^*2*^ = 93.3%).

### 3.10 Heterogeneity and Bias Assessment

Substantial heterogeneity was observed across the 85 included effect sizes. The random‐effects model estimated a between‐study variance (τ^2^) of 1.82 (SE = 0.29), with *I* ^2^ = 97.08% and H^2^ = 34.21. These values suggest that nearly all observed variability is due to true differences between studies rather than random sampling error. The Q‐statistic for heterogeneity was significant (Q(84) = 1431.20, *p* < 0.001), indicating considerable variability in effect sizes across trials. To evaluate potential publication bias, we applied the trim‐and‐fill method. No studies were imputed on the right side (SE = 1.99), suggesting that missing studies with null or positive effects were unlikely and that the pooled result was robust to potential bias from unpublished trials (Supporting Information S1: eFigure 2B). However, Egger”s regression test indicated significant funnel plot asymmetry (*z* = −6.28, *p* < 0.001) (Supporting Information S1: eFigure 2A), suggesting the presence of small‐study effects or methodological differences. Given the high heterogeneity, we conducted additional sensitivity analyses. A leave‐one‐out analysis confirmed that no individual study unduly influenced the overall result: removing any single effect size shifted the pooled estimate only slightly, ranging from *g* = −1.05 to *g* = −0.96, all with *p* < 0.001 (Supporting Information S1: eFigure 4). Visual inspection of the leave‐one‐out plot supports the stability of the pooled estimate. Influence diagnostics (e.g., Baujat plots) indicated that while a few studies contributed more strongly to heterogeneity or effect size, none significantly distorted the overall results (Supporting Information S1: eFigure 4).

We also explored risk of bias as a potential modifier. Using the Cochrane Risk of Bias Tool 2.0, we evaluated each study across five domains: randomization, deviations from intended interventions, missing data, outcome measurement, and reporting bias. Most studies were rated low risk, although some showed concerns or high risk in domains such as randomization and outcome measurement (Supporting Information S1: eFigure 4). A sensitivity analysis excluding high‐risk studies yielded a similar pooled effect size (*g* = −0.96), reinforcing the robustness of findings. While these diagnostics support the reliability of the meta‐analytic results, we acknowledge that funnel plots, Egger”s test, and trim‐and‐fill may be unstable under high heterogeneity and dependent effect sizes. Selection models were considered but not implemented due to limited power. Overall, the pattern of findings emphasizes the need for larger, well‐controlled, and transparently reported trials to improve precision and reduce bias in future MBI research.

## 4 Discussion

In this meta‐analysis of 45 randomized controlled trials involving 7395 adults with cancer, we found that mindfulness‐based interventions (MBIs) significantly reduced symptoms of depression, anxiety, and stress. The strongest effects were observed for stress, and our findings suggest that these improvements are clinically meaningful. We observed the largest effects from adapted or modified MBIs, including brief, digital, or oncology‐specific formats, followed by MBSR and MBCT. Participants with breast cancer experienced the most benefit, although we found positive effects across various cancer types. Our results also indicated that programs with shorter contact hours, longer durations (8 weeks or more), and lower dropout rates were associated with stronger outcomes. Using a multilevel meta‐analysis with robust variance estimation, we accounted for dependencies among effect sizes and found a pooled effect size of *g* = −0.88. In meta‐regression, we identified that interventions targeting both depression and anxiety yielded significantly greater effects than those focused on a single domain. To support interpretation, we translated standardized effect sizes into estimated raw score reductions, which further confirmed the clinical relevance of our findings. While heterogeneity was high, our analysis supports MBIs as effective, scalable strategies to improve psychological well‐being in cancer care.

These results align with previous findings [22, 24, 38], while offering additional comparisons by intervention type, cancer group, and region. Unlike prior reviews, this study reports clinically interpretable effect sizes and stratified subgroup results. MBIs such as Mindfulness‐Based Stress Reduction (MBSR) and Mindfulness‐Based Cognitive Therapy (MBCT) have shown efficacy in alleviating distress in cancer populations [10, 30, 39]. However, past meta‐analyses often combined diverse outcomes or lacked differentiation by cancer type or MBI format [22, 23, 24, 25, 39, 40].

This study extends prior work by showing that adapted or brief MBIs may be more effective than standard programs, particularly for stress. Breast cancer studies consistently reported the strongest effects, consistent with previous findings [31, 41, 42]. Stratified analyses revealed comparable benefits across global regions, confirming the generalizability of MBIs. These findings highlight the adaptability of MBIs in cancer care and emphasize the potential value of brief and digital delivery models [43, 44].

### 4.1 Clinical Implications

This review supports the use of mindfulness‐based interventions (MBIs) as an effective approach for improving psychological outcomes in adults with cancer. Findings indicate that MBIs are associated with meaningful reductions in depression, anxiety, and stress symptoms, with consistent benefits across diverse cancer populations. Given their flexibility and low risk, MBIs are well suited for integration into psychosocial oncology care. Modified MBIs formats, including brief or digitally delivered programs, showed strong effects and may be particularly beneficial in settings with limited access to traditional mental health services. Implementation strategies should prioritize accessibility and acceptability. Delivering MBIs in group‐based or remote formats may help expand reach, especially in resource‐constrained or rural settings. Tailoring interventions to specific patient populations, such as breast cancer survivors, may enhance effectiveness, as suggested by subgroup findings. While results are encouraging, further research is needed to explore long‐term outcomes, optimal delivery formats, and adaptation across cancer types. Nonetheless, the current evidence highlights MBIs as a valuable component of supportive care in oncology.

### 4.2 Limitations

Several subgroup analyses were based on a small number of studies, especially for certain cancer types and intervention formats, which may reduce estimate stability and increase susceptibility to small‐study effects. Despite stratification by mental health outcome, MBI type, and population, heterogeneity remained high, reflecting variations in intervention design, duration, and participant characteristics.

The analysis relied on study‐level data, limiting the ability to adjust for individual‐level factors such as baseline symptom severity, treatment phase, or comorbidities. While Egger”s test indicated potential publication bias, sensitivity analyses, including trim‐and‐fill, suggested that the overall findings remained robust. Nevertheless, variability in outcome measures and follow‐up durations limits conclusions about the long‐term effects of MBIs. Reporting of implementation factors was also limited across trials. Instructor qualifications, fidelity procedures, and participant adherence (e.g., session attendance or home practice) were inconsistently reported, and adverse events or harms were rarely monitored or documented.

Generalizability is further constrained by the overrepresentation of women with breast cancer. Few studies included male participants or individuals with non‐breast cancers, and reporting on cancer stage or treatment status was often incomplete. These limitations restrict the applicability of findings to more diverse oncology populations. Reporting of dropout and adherence was inconsistent. While most studies reported attrition below 20%, several exceeded this level, often without specifying timing or reasons. Adherence data and instructor qualifications were rarely reported, limiting further analysis. Few studies mentioned adverse events, and none reported intervention‐related harm. Although MBIs appear safe and acceptable, more rigorous reporting is needed to evaluate adherence, fidelity, and potential risks. Despite these challenges, the consistent pattern of psychological benefit observed across trials highlights the promise of MBIs as a supportive care strategy in cancer settings. With broader inclusion and improved methodological reporting, future research can further strengthen the evidence base and expand access to effective mind‐body interventions.

### 4.3 Future Research

Future trials should evaluate the effectiveness of MBIs in underrepresented cancer types such as lung and prostate, and in more demographically diverse populations. Long‐term outcomes, cost‐effectiveness, and mechanisms of action also warrant further investigation. Consistent and standardized reporting of depression, anxiety, and stress outcomes is critical to improve comparability across studies. While we did not conduct a subgroup analysis comparing passive control conditions (such as waitlist or treatment‐as‐usual) with active comparators, this should be prioritized in future work, as comparator type can influence effect sizes. Despite evidence of small‐study effects and substantial heterogeneity, sensitivity analyses including trim‐and‐fill, leave‐one‐out, and influence diagnostics suggest that the overall findings are robust. However, future trials should aim for greater methodological consistency and transparency to reduce bias and improve interpretability. Addressing these gaps will help refine implementation strategies and strengthen the evidence base supporting MBIs as an accessible and scalable approach to improving mental health in oncology care.

## 5 Conclusion

Mindfulness‐based interventions were associated with reduced depression, anxiety, and stress in adults with cancer. However, heterogeneity was high, most trials involved breast cancer survivors, and comparisons across MBI types were indirect. Adverse events were infrequently reported, limiting conclusions about potential risks. Broader generalization across cancer types remains premature. These findings support MBIs as part of tailored psychosocial care while underscoring the need for more diverse and rigorously controlled trials.

## Funding

Open access funding was provided by North‐West University, South Africa.

## Ethics Statement

The authors have nothing to report.

## Consent

The authors have nothing to report.

## Conflicts of Interest

The authors declare no conflicts of interest.

## Supporting information


Supporting Information S1

## Data Availability

The data that support the findings of this study are available on request from the corresponding author. The data are not publicly available due to privacy or ethical restrictions.

## References

[1] F. Bray , M. Laversanne , H. Sung , et al., “Global Cancer Statistics 2022: GLOBOCAN Estimates of Incidence and Mortality Worldwide for 36 Cancers in 185 Countries,” CA: A Cancer Journal for Clinicians 74, no. 3 (2024): 229–263, 10.3322/caac.21834. 38572751

[2] J. Ferlay , M. Ervik , F. Lam , et al., “Global Cancer Observatory: Cancer Today,” International Agency for Research on Cancer (2020): 20182020.

[3] S. Habimana , E. Biracyaza , T. Mpunga , et al., “Prevalence and Associated Factors of Depression and Anxiety Among Patients With Cancer Seeking Treatment at the Butaro Cancer Center of Excellence in Rwanda,” Frontiers in Public Health 11 (2023): 972360, 10.3389/fpubh.2023.972360. 36875374 PMC9978744

[4] D. Miro‐Rivera , R. A. Norris , O. L. Osazuwa‐Peters , J. H. Hurst , J. M. Barnes , and N. Osazuwa‐Peters , “Differential Use of Depression and Anxiety Medications in Adults With a History of Cancer,” JAMA Network Open 8, no. 8 (2025): e2527585, 10.1001/jamanetworkopen.2025.27585. 40828535 PMC12365704

[5] R. Y. Soong , C. E. Low , V. Ong , et al., “Exercise Interventions for Depression, Anxiety, and Quality of Life in Older Adults With Cancer: A Systematic Review and Meta‐Analysis,” JAMA Network Open 8, no. 2 (2025): e2457859, 10.1001/jamanetworkopen.2024.57859. 39903465 PMC11795328

[6] P. Y. Yu , F. Liu , Y. Jiao , Y. C. Zhao , and Y. H. Liu , “Depression in Gastric Cancer Patients: Integrated Therapeutic Strategies and Clinical Implications,” World Journal of Clinical Oncology 16, no. 6 (2025): 106229, 10.5306/wjco.v16.i6.106229. 40585841 PMC12198864

[7] E. Arden‐Close , F. Mitchell , G. Davies , et al., “Mindfulness‐Based Interventions in Recurrent Ovarian Cancer: A Mixed‐Methods Feasibility Study,” Integrative Cancer Therapies 19 (2020): 1534735420908341, 10.1177/1534735420908341. 32174190 PMC7076576

[8] H. M. Dragomanovich , A. Dhruva , E. Ekman , et al., “Being Present 2.0: Online Mindfulness‐Based Program for Metastatic Gastrointestinal Cancer Patients and Caregivers,” Global Advances in Health and Medicine 10 (2021): 21649561211044693, 10.1177/21649561211044693. 35174001 PMC8842457

[9] M. Price‐Blackshear , S. Pratscher , D. Oyler , et al., “Online Couples Mindfulness‐Based Intervention for Young Breast Cancer Survivors and Their Partners: A Randomized‐Control Trial,” Journal of Psychosocial Oncology 38, no. 5 (2020): 592–611, 10.1080/07347332.2020.1778150. 32552446

[10] Y. L. Wang , X. F. Zhang , X. P. Wang , Y. J. Zhang , Y. Y. Jin , and W. L. Li , “Combined Mindfulness‐Based Stress Reduction and Exercise Intervention for Improving Psychological Well‐Being in Patients With Non‐Small Cell Lung Cancer,” Clinical Psychology and Psychotherapy 31, no. 4 (2024): e3023, 10.1002/cpp.3023. 38978207

[11] Z. Xu , C. Liu , W. Fan , S. Li , and Y. Li , “Effect of Music Therapy on Anxiety and Depression in Breast Cancer Patients: Systematic Review and Meta‐Analysis,” Scientific Reports 14, no. 1 (2024): 16532, 10.1038/s41598-024-66836-x. 39019965 PMC11255342

[12] C. Barrios , G. de Lima Lopes , M. M. Yusof , F. Rubagumya , P. Rutkowski , and M. Sengar , “Barriers in Access to Oncology Drugs — A Global Crisis,” Nature Reviews Clinical Oncology 20, no. 1 (2023): 7–15, 10.1038/s41571-022-00700-7. PMC9665041 36380066

[13] M. Juarez‐Reyes , N. Purington , and S. Kling , “Mindfulness‐Based Group Medical Visits in Primary Care for Stress and Anxiety: An Observational Study,” J Integr Complement Med 28, no. 9 (2022): 721–728, 10.1089/jicm.2021.0329. 35671517

[14] M. Juarez‐Reyes , E. Martinez , L. Xiao , and L. Goldman Rosas , “A Randomized Controlled Trial of a Culturally Modified MBIs, Community‐Based, Remotely Delivered Mindfulness Program for Latinx Patients With Breast Cancer is Acceptable and Feasible While Reducing Anxiety,” Glob Adv Integr Med Health 13 (2024): 27536130241274240, 10.1177/27536130241274240. 39157776 PMC11329901

[15] R. Crane , J. Brewer , C. Feldman , et al., “What Defines Mindfulness‐Based Programs? the Warp and the Weft,” Psychological Medicine 47, no. 6 (2017): 990–999, 10.1017/S0033291716003317. 28031068

[16] S. Guendelman , M. Bayer , K. Prehn , and I. Dziobek , “Towards a Mechanistic Understanding of Mindfulness‐Based Stress Reduction (MBSR) Using an RCT Neuroimaging Approach: Effects on Regulating Own Stress in Social and Non‐Social Situations,” NeuroImage 254 (2022): 119059, 10.1016/j.neuroimage.2022.119059. 35259523

[17] J. Kabat‐Zinn , “Mindfulness‐Based Stress Reduction (MBSR),” Constructivism in the Human Sciences 8, no. 2 (2003): 73.

[18] Z. Segal , M. Williams , and J. Teasdale , Mindfulness‐Based Cognitive Therapy for Depression Guilford press, (2012).

[19] J. Tatta , A. M. Willgens , and K. M. Palombaro , “Mindfulness and Acceptance–Based Interventions in Physical Therapist Practice: The Time is now,” Physical Therapy 102, no. 3 (2022): pzab293, Accessed, June 14, 2024, https://academic.oup.com/ptj/article‐abstract/102/3/pzab293/6481182. 35079796 10.1093/ptj/pzab293

[20] L. Russell , A. Ugalde , L. Orellana , et al., “A Pilot Randomised Controlled Trial of an Online Mindfulness‐Based Program for People Diagnosed With Melanoma,” Supportive Care in Cancer 27, no. 7 (2019): 2735–2746, 10.1007/s00520-018-4574-6. 30506103

[21] F. Xu , J. Zhang , S. Xie , and Q. Li , “Effects of Mindfulness‐Based Cancer Recovery Training on Anxiety, Depression, Post‐Traumatic Stress Disorder, and Cancer‐Related Fatigue in Breast Neoplasm Patients Undergoing Chemotherapy,” Med Baltim 103, no. 23 (2024): e38460, 10.1097/MD.0000000000038460. PMC11155580 38847730

[22] N. Badaghi , C. Buskbjerg , L. Kwakkenbos , S. Bosman , R. Zachariae , and A. Speckens , “Positive Health Outcomes of Mindfulness‐Based Interventions for Cancer Patients and Survivors: A Systematic Review and Meta‐Analysis,” Clinical Psychology Review 114 (2024): 102505, 10.1016/j.cpr.2024.102505. 39316940

[23] L. Y. Lin , L. H. Lin , G. L. Tzeng , et al., “Effects of Mindfulness‐Based Therapy for Cancer Patients: A Systematic Review and Meta‐Analysis,” Journal of Clinical Psychology in Medical Settings 29, no. 2 (2022): 432–445, 10.1007/s10880-022-09862-z. 35249176

[24] S. Oberoi , J. Yang , R. L. Woodgate , et al., “Association of Mindfulness‐Based Interventions With Anxiety Severity in Adults With Cancer: A Systematic Review and Meta‐Analysis,” JAMA Network Open 3, no. 8 (2020): e2012598, 10.1001/jamanetworkopen.2020.12598. 32766801 PMC7414391

[25] E. Štánerová , V. Zelenayová , and J. Rajčáni , “Mindfulness‐Based Interventions for Cancer Patients in Standard Treatment: A Meta‐Analysis of Effects on Depression, Anxiety, and Quality of Life,” Journal of Psychosomatic Research 196 (2025): 112312, 10.1016/j.jpsychores.2025.112312. 40639226

[26] M. J. Page , J. E. McKenzie , P. M. Bossuyt , et al., “The PRISMA 2020 Statement: An Updated Guideline for Reporting Systematic Reviews,” Bmj 372 (2021), 10.1136/bmj.n71. PMC8005924 33782057

[27] J. A. Sterne , J. Savović , M. J. Page , et al., “Rob 2: A Revised Tool for Assessing Risk of Bias in Randomised Trials,” BMJ 366 (2019), 10.1136/bmj.l4898. 31462531

[28] M. Egger , G. Davey Smith , M. Schneider , and C. Minder , “Bias in Meta‐Analysis Detected by a Simple, Graphical Test,” BMJ 315, no. 7109 (1997): 629–634, 10.1136/bmj.315.7109.629. 9310563 PMC2127453

[29] M. Mirmahmoodi , P. Mangalian , A. Ahmadi , and M. Dehghan , “The Effect of Mindfulness‐Based Stress Reduction Group Counseling on Psychological and Inflammatory Responses of the Women With Breast Cancer,” Integrative Cancer Therapies 19 (2020): 1534735420946819, 10.1177/1534735420946819. 33078649 PMC7594228

[30] R. Nissim , A. Roth , A. Gupta , and M. Elliott , “Mindfulness‐Based Cognitive Therapy Intervention for Young Adults With Cancer: A Pilot Mixed‐Method Study,” Journal of Adolescent and Young Adult Oncology 9, no. 2 (2020): 256–261, 10.1089/jayao.2019.0086. 31621473

[31] S. Park , Y. Sato , Y. Takita , et al., “Mindfulness‐Based Cognitive Therapy for Psychological Distress, Fear of Cancer Recurrence, Fatigue, Spiritual Well‐Being, and Quality of Life in Patients With Breast Cancer—A Randomized Controlled Trial,” Journal of Pain and Symptom Management 60, no. 2 (2020): 381–389, 10.1016/j.jpainsymman.2020.02.017. 32105790

[32] D. Messer , J. Horan , L. Larkey , and C. Shanholtz , “Effects of Internet Training in Mindfulness Meditation on Variables Related to Cancer Recovery,” Mindfulness 10, no. 10 (2019): 2143–2151, 10.1007/s12671-019-01182-y.

[33] Lengacher C. , Reich R. , Paterson C. , et al. “Examination of Broad Symptom Improvement Resulting From Mindfulness‐Based Stress Reduction in Breast Cancer Survivors: A Randomized Controlled Trial.” Journal of Clinical Oncology. (2016); 34(24):2827–2834, 10.1200/JCO.2015.65.7874 27247219 PMC5012660

[34] P. A. Cohen , T. Musiello , S. Jeffares , and K. Bennett , “Mindfulness‐Based Cognitive Therapy for Fear of Recurrence in Ovarian Cancer Survivors (FROCS): A Single‐Arm, Open‐Label, Pilot Study,” Supportive Care in Cancer 30, no. 3 (2022): 2317–2325, 10.1007/s00520-021-06659-y. 34727225

[35] M. Wirth , R. Franco , S. Robb , et al., “Randomized Controlled Trial of a 4‐Week Mindfulness Intervention Among Cancer Survivors Compared to a Breathing Control,” Cancer Investigation 37, no. 4–5 (2019): 227–232, 10.1080/07357907.2019.1610968. 31198066

[36] K. Cavanagh , C. Strauss , F. Cicconi , N. Griffiths , A. Wyper , and F. Jones , “A Randomised Controlled Trial of a Brief Online Mindfulness‐based Intervention,” Behaviour Research and Therapy 51, no. 9 (2013): 573–578, 10.1016/j.brat.2013.06.003. 23872699

[37] R. Bränström , P. Kvillemo , Y. Brandberg , and J. T. Moskowitz , “Self‐Report Mindfulness as a Mediator of Psychological Well‐Being in a Stress Reduction Intervention for Cancer patients—A Randomized Study,” Annals of Behavioral Medicine 39, no. 2 (2010): 151–161, 10.1007/s12160-010-9168-6. 20177843

[38] N. Z. Zainal , S. Booth , and F. A. Huppert , “The Efficacy of Mindfulness‐Based Stress Reduction on Mental Health of Breast Cancer Patients: A Meta‐Analysis,” Psycho‐Oncology 22, no. 7 (2013): 1457–1465, 10.1002/pon.3171. 22961994

[39] L. Cillessen , M. Johannsen , A. E. M. Speckens , and R. Zachariae , “Mindfulness‐Based Interventions for Psychological and Physical Health Outcomes in Cancer Patients and Survivors: A Systematic Review and Meta‐Analysis of Randomized Controlled Trials,” Psycho‐Oncology 28, no. 12 (2019): 2257–2269, 10.1002/pon.5214. 31464026 PMC6916350

[40] J. Piet , H. Würtzen , and R. Zachariae , “The Effect of Mindfulness‐Based Therapy on Symptoms of Anxiety and Depression in Adult Cancer Patients and Survivors: A Systematic Review and Meta‐Analysis,” Journal of Consulting and Clinical Psychology 80, no. 6 (2012): 1007–1020, 10.1037/a0028329. 22563637

[41] C. Lengacher , R. Reich , C. Paterson , et al., “The Effects of Mindfulness‐Based Stress Reduction on Objective and Subjective Sleep Parameters in Women With Breast Cancer: A Randomized Controlled Trial,” Psycho‐Oncology 24, no. 4 (2015): 424–432, 10.1002/pon.3603. 24943918 PMC4487655

[42] C. Lengacher , R. Reich , C. Rodriguez , et al., “Efficacy of Mindfulness‐Based Stress Reduction for Breast Cancer (MBSR(BC)) a Treatment for Cancer‐Related Cognitive Impairment (CRCI): A Randomized Controlled Trial,” J Integr Complement Med 31, no. 1 (2025): 75–91, 10.1089/jicm.2024.0184. 39291332 PMC11839530

[43] F. Bruggeman‐Everts , M. Wolvers , R. Van de Schoot , M. Vollenbroek‐Hutten , and M. Van der Lee , “Effectiveness of Two Web‐Based Interventions for Chronic Cancer‐Related Fatigue Compared to an Active Control Condition: Results of the ‘Fitter Na Kanker’ Randomized Controlled Trial,” Journal of Medical Internet Research 19, no. 10 (2017): e336, 10.2196/jmir.7180. 29051138 PMC5668634

[44] S. A. Johns , K. Kroenke , E. E. Krebs , D. E. Theobald , J. Wu , and W. Tu , “Longitudinal Comparison of Three Depression Measures in Adult Cancer Patients,” Journal of Pain and Symptom Management 45, no. 1 (2013): 71–82, 10.1016/j.jpainsymman.2011.12.284. 22921152 PMC3538946

