# Supplementary material for: Mindfulness‐Based Interventions for Depression, Anxiety, and Stress in Adults With Cancer: A Stratified Subgroup Meta‐Analysis
Source: Psychooncology. 2026 Mar 12;35(3):e70424. doi: 10.1002/pon.70424 (PMC12982628; doi:10.1002/pon.70424)
Supplement: Supplementary file 1 — Supporting Information S1 [file PON-35-e70424-s001.docx]

**Supplemental Online Content**

**eMethods Table 1. Search Strategy and Keywords**

| **Concept** | **Search Terms (with synonyms and truncations)** |
| --- | --- |
| 1. Mindfulness-based Interventions | “mindfulness” OR “mindfulness-based” OR “mindfulness-based stress reduction” OR “MBSR” OR “mindfulness-based cognitive therapy” OR “MBCT” OR “mindfulness training” OR “meditation” |
| 2. Psychological Outcomes | “depression” OR “depressive symptoms” OR “anxiety” OR “stress” OR “psychological distress” OR “mental health” |
| 3. Cancer Population | “cancer” OR “oncology” OR “tumor” OR “carcinoma” OR “neoplasm” OR “malignancy” OR “cancer survivor*” |
| 4. Study Design | “randomized controlled trial” OR “RCT” OR “randomised controlled trial” OR “randomized trial” |

Combined Boolean Search String: TS=(“mindfulness” OR “mindfulness-based” OR “MBSR” OR “MBCT” OR “mindfulness training” OR “meditation”) AND TS=(“depression” OR “anxiety” OR “stress” OR “psychological distress” OR “mental health”) AND TS=(“cancer” OR “oncology” OR “tumor” OR “neoplasm” OR “malignancy” OR “cancer survivor*”) AND TS=(“randomized controlled trial” OR “RCT” OR “randomised controlled trial”)

1. Web of Science (WoS): TS=(“mindfulness” OR “mindfulness-based” OR “MBSR” OR “MBCT” OR “mindfulness training” OR “meditation”) AND TS=(“depression” OR “anxiety” OR “stress” OR “psychological distress” OR “mental health”) AND TS=(“cancer” OR “oncology” OR “tumor” OR “neoplasm” OR “malignancy” OR “cancer survivor*”) AND TS=(“randomized controlled trial” OR “RCT” OR “randomised controlled trial”)
2. **Scopus:** TITLE-ABS-KEY(“mindfulness” OR “mindfulness-based” OR “MBSR” OR “MBCT” OR “meditation”) AND TITLE-ABS-KEY(“depression” OR “anxiety” OR “stress” OR “psychological distress” OR “mental health”) AND TITLE-ABS-KEY(“cancer” OR “oncology” OR “tumor” OR “neoplasm” OR “malignancy” OR “cancer survivor*”) AND TITLE-ABS-KEY(“randomized controlled trial” OR “RCT” OR “randomised controlled trial”)
3. ScienceDirect: (“mindfulness” OR “MBSR” OR “MBCT” OR “mindfulness-based”) AND (“depression” OR “anxiety” OR “stress” OR “mental health”) AND (“cancer” OR “oncology” OR “neoplasm”) AND (“randomized controlled trial” OR “RCT”)
4. EBSCOhost (e.g., APA PsycInfo, MEDLINE, CINAHL): (mindfulness OR “mindfulness-based stress reduction” OR MBSR OR MBCT OR meditation) AND (depression OR anxiety OR stress OR “psychological distress”) AND (cancer OR oncology OR neoplasm OR malignancy OR “cancer survivor*”) AND (“randomized controlled trial” OR RCT)

**eFigure 1. Risk of Bias Assessment**


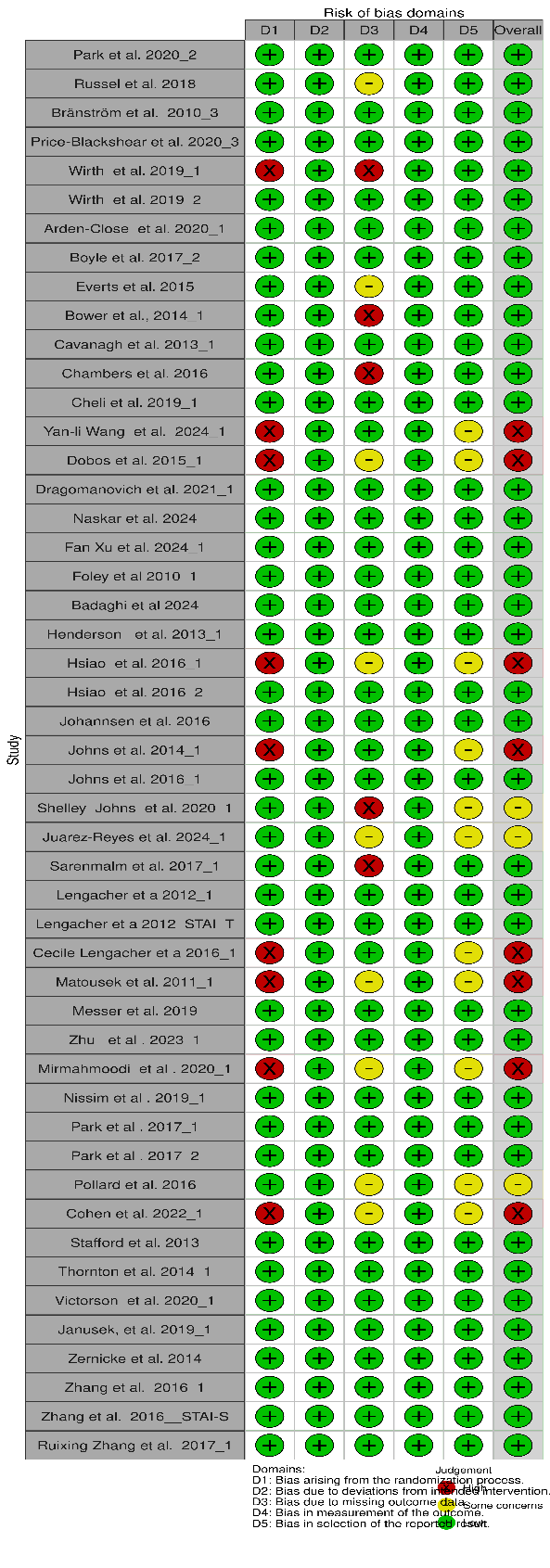


**eFigure 2. Funnel Plots of Publication Bias for Overall Effects**

(A) Original funnel plot showing asymmetry in the distribution of effect sizes. (B) Funnel plot with trim-and-fill adjustment indicating potential missing studies. Standard error is plotted against standardized mean difference (Hedges’ *g*).

**eFigure 3.** Clinical Interpretation of Effect Sizes

**eFigure 4.** Influence and Outlier Diagnostics and Sensitivity Analysis

**Panel (a).** Leave-one-out sensitivity analysis. Each dot represents the pooled effect size with one study omitted. The minimal variation demonstrates the stability of the findings.

**Panel (b).** Influence plot showing squared Pearson residuals vs. influence on the overall result. Study 8 shows notably high influence.

**Panel (c).** Diagnostic plots (e.g., studentized residuals, Cook's D, DFFITS, heterogeneity estimates) to identify influential and potentially problematic studies.

**eTable 2. Characteristics of the Included Studies**

| **Study (et al.)** | **Age (mean ± SD)** | **Gender (Male/Female)** | Number of Dropouts (%) | **Outcomes Measure** | **Variables(depression, anxienty or stress)** | **Cancer Population** | **MBIs** |
| --- | --- | --- | --- | --- | --- | --- | --- |
| Park et al. 2020^1^ | 53.7 ± 8.8 | 74 (100% fmale) | 3 | Hospital Anxiety and Depression Scale (HADS) | Anxiety | Breast Cancer | Mindfulness-Based Cognitive Therapy (MBCT) |
| Park et al. 2020_2 | 53.7 ± 8.9 | 75 (100% fmale) | 3 | Hospital Anxiety and Depression Scale (HADS) | Depression | Breast Cancer | Mindfulness-Based Cognitive Therapy (MBCT) |
| Russel et al. 2019^2^ | 53.4 ± 13.1 | 32/37 (46% male / 54% female) | 7 | Perceived Stress Scale (PSS-10) | Stress | melanoma cancer | Online mindfulness-based Intervention (iMBI) |
| Bränström et al. 2010^3^ | 51.8±9.8 | 1=male/ 70=female | np | Perceived Stress Scale (PSS) | Stress | Cancer Survivors | Mindfulness-Based Stress Reduction (MBSR) |
| Bränström et al. 2010 | 51.8±9.9 | 1=male/ 70=female | np | Hospital Anxiety and Depression Scale (HADS) | Anxiety | Cancer Survivors | Mindfulness-Based Stress Reduction (MBSR) |
| Bränström et al. 2010 | 51.8±9.10 | 1=male/ 70=female | np | Hospital Anxiety and Depression Scale (HADS) | Depression | Cancer Survivors | Mindfulness-Based Stress Reduction (MBSR) |
| Price-Blackshear et al. 2020^4^ | 38.78 ± 5.08 | Female | 0.26 | Perceived Stress Scale (PSS) | Stress | Breast Cancer | mindfulness-based intervention (MBI) |
| Price-Blackshear et al. 2020 | 38.78 ± 5.09 | Female | 1.26 | PROMIS Anxiety short forms | Anxiety | Breast Cancer | mindfulness-based intervention (MBI) |
| Price-Blackshear et al. 2020 | 38.78 ± 5.10 | Female | 2.26 | PROMIS Depression short forms | Depression | Breast Cancer | mindfulness-based intervention (MBI) |
| Wirth et al. 2019^5^ | 63.9 ± 10.1 yrs | Female: 72%, Male: 28% (N=36; 19 breast, 7 prostate, 3 gynecologic, others mixed) | 0.1 | PSS (Perceived Stress Scale) | Stress | Cancer Survivors | mindfulness-based cancer survivorship (MBCS) |
| Wirth et al. 2019 | 63.9 ± 10.1 yrs | Female: 72%, Male: 28% (N=36; 19 breast, 7 prostate, 3 gynecologic, others mixed) | 0.1 | CES-D (Depression) | Depression | Cancer Survivors | mindfulness-based cancer survivorship (MBCS) |
| Arden-Close et al. 2020^6^ | 59 ± 10 years | Female: 100% (N = 28) | 0.036 | HADS (Hospital Anxiety and Depression Scale) | Anxiety | Ovarian Cancer | mindfulness-based intervention (MBI) |
| Arden-Close et al. 2020 | 60 ± 10 years | Female: 100% (N = 28) | 1.036 | HADS (Hospital Anxiety and Depression Scale) | Depression | Ovarian Cancer | mindfulness-based intervention (MBI) |
| Boyle et al. 2017^7^ | Mean age = 47 (range 28–60) | Female: 100% (N = 71) | 0.17 | PSS (Perceived Stress Scale) | Stress | Breast Cancer | mindfulness-based intervention (MBI) |
| Boyle et al. 2017 | Mean age = 47 (range 28–60) | Female: 100% (N = 71) | 1.17 | CES-D (Center for Epidemiologic Studies Depression Scale) | Depression | Breast Cancer | mindfulness-based intervention (MBI) |
| Everts et al. 2015^8^ | 50.2 ± 10.7 years | Female: 76.3% (N ≈ 196), Male: 23.7% (N ≈ 61) | 0.381 | Hospital Anxiety and Depression Scale (HADS) | Anxiety and Depression | Cancer Survivors | Web-based individual Mindfulness-Based Cognitive Therapy |
| Bower et al., 2014^7^ | 46.1 years (range 28–60) | Female: 100% | 0.11 | Perceived Stress Scale (PSS) | Stress | Breast Cancer | mindfulness-based intervention (MBI) |
| Bower et al., 2014 | 46.1 years (range 28–60) | Female: 100% | 1.11 | CES-D (Center for Epidemiologic Studies Depression Scale) | Depression | Breast Cancer | mindfulness-based intervention (MBI) |
| Cavanagh et al. 201^9^ | 24.7 ± 6.44 years (range 19–51) | Female: 88.5% (N=92), Male: 11.5% (N=12) | 57.4 | Perceived Stress Scale (PSS) | Anxiety | Prostate Cancer | mindfulness-based cognitive therapy (MBCT) |
| Cavanagh et al. 2013 | 24.7 ± 6.44 years (range 19–51) | Female: 88.5% (N=92), Male: 11.5% (N=12) | 58.4 | PHQ-4 (Patient Health Questionnaire for Depression & Anxiety) | Depression | Prostate Cancer | mindfulness-based cognitive therapy (MBCT) |
| Chambers et al. 2017^10^ | MBCT: 70.2 ± 8.7 yrs; Control: 71.2 ± 8.4 yrs | Male: 100% (N = 189; 94 MBCT, 95 control) | 0.14 | Memorial Anxiety Scale for Prostate Cancer (PSA anxiety) | Anxiety | Prostate Cancer | mindfulness-based cognitive therapy (MBCT) |
| Cheli et al. 2019^11^ | 53.4 ± 9.1 years (overall); Group: 54.5 ± 7.8, Individual: 52.9 ± 9.7 | Female: 100% (N = 114; Group = 38, Individual = 76) | 0.07 | HADS (Hospital Anxiety & Depression Scale) | Anxiety | Breast Cancer | mindfulness-based intervention (MBI) |
| Cheli et al. 2019 | 53.4 ± 9.1 years (overall); Group: 54.5 ± 7.8, Individual: 52.9 ± 9.8 | Female: 100% (N = 114; Group = 38, Individual = 76) | 0.07 | HADS (Hospital Anxiety & Depression Scale) | Depression | Breast Cancer | mindfulness-based intervention (MBI) |
| Yan-li Wang et al. 2024^12^ | Intervention: 54.44 ± 8.07; Control: 54.40 ± 9.96 | Male: 20 (66.7%), Female: 10 (33.3%) in each group (N = 60 total) | N/A | SAS (Self-Rating Anxiety Scale) | Anxiety | Cell Lung Cancer | mindfulness-based stress reduction (MBSR) |
| Yan-li Wang et al. 2024 | Intervention: 54.44 ± 8.07; Control: 54.40 ± 9.97 | Male: 20 (66.7%), Female: 10 (33.3%) in each group (N = 60 total) | N/A | SDS (Self-Rating Depression Scale) | Depression | Cell Lung Cancer | mindfulness-based stress reduction (MBSR) |
| Dobos et al. 2015^13^ | 53.9 ± 10.7 years (range 24–77) | Female: 91% (N = 106), Male: 9% (N = 11) | 0.162 | Hospital Anxiety and Depression Scale (HADS) | Anxiety | Cancer Survivors | mindfulness-based stress reduction (MBSR) |
| Dobos et al. 2015 | 53.9 ± 10.7 years (range 24–77) | Female: 91% (N = 106), Male: 9% (N = 11) | 0.162 | Hospital Anxiety and Depression Scale (HADS) | Depression | Cancer Survivors | mindfulness-based stress reduction (MBSR) |
| Dragomanovich et al. 2021^14^ | Median = 52 years (range 20–70) | Male: 18 (39%), Female: 28 (61%) (N = 46 patients) | 0.17 | PROMIS Anxiety | Anxiety | Metastatic Cancer | mindfulness-based intervention (MBI) |
| Dragomanovich et al. 2021 | Median = 52 years (range 20–70) | Male: 18 (39%), Female: 28 (61%) (N = 46 patients) | 0.17 | PROMIS Depression | Depression | Metastatic Cancer | mindfulness-based intervention (MBI) |
| Naskar et al. 2024^15^ | Intervention: 56.25 ± 9.59; Control: 51.05 ± 11.27 | Female: 100% (N = 40; 20 intervention, 20 control) | N/A | Perceived Stress Scale (PSS-10) | Stress | Breast Cancer | mindfulness-based intervention (MBI) |
| Fan Xu et al. 2024^16^ | Study: 53.18 ± 14.39 (range 27–78); Control: 52.88 ± 12.61 (range 26–75) | Female: 100% (N = 80; 40 intervention, 40 control) | N/A | HADS (Hospital Anxiety and Depression Scale) | Anxiety | Breast Cancer | Mindfulness-Based Cancer Recovery (MBCR) |
| Fan Xu et al. 2024 | Study: 53.18 ± 14.39 (range 27–78); Control: 52.88 ± 12.61 (range 26–75) | Female: 100% (N = 80; 40 intervention, 40 control) | N/A | HADS (Hospital Anxiety and Depression Scale) | Depression | Breast Cancer | Mindfulness-Based Cancer Recovery (MBCR) |
| Foley et al 2010^17^ | 55.18 ± 10.60 years (range 24–78) | Female: 77% (N = 89), Male: 23% (N = 26); N = 115 total | 0.07 | , Hamilton Anxiety Rating Scale (HAM-A) | Anxiety | Cancer | Mindfulness based cognitive therapy (MBCT) |
| Foley et al 2010 | 55.18 ± 10.60 years (range 24–78) | Female: 77% (N = 89), Male: 23% (N = 26); N = 115 total | 0.07 | Hamilton Depression Rating Scale (HAM-D) | Depression | Cancer | Mindfulness based cognitive therapy (MBCT) |
| Badaghi et al 2024^18^ | Mean = 51 ± 11.5 years | Female: 87% (N = 21), Male: 13% (N = 3), N = 24 | 0.42 | HADS (Hospital Anxiety and Depression Scale) | Anxiety and Depression | Cancer | Mindfulness based cognitive therapy (MBCT) |
| Henderson et al. 2013^19^ | Mean ≈ 50 ± 8 years | Female: 100% (N = 172 randomized; 120 received radiotherapy) | 0.044 | Beck Anxiety Inventory | Anxiety | Breast Cancer | mindfulness-based stress-reduction (MBSR) |
| Henderson et al. 2013 | Mean ≈ 50 ± 8 years | Female: 100% (N = 172 randomized; 120 received radiotherapy) | 0.044 | Beck Depression Inventory | Depression | Breast Cancer | mindfulness-based stress-reduction (MBSR) |
| Hsiao et al. 2016^20^ | 18–65 years | Female: 100% of survivors | 0.075 | State-Trait Anxiety Inventory (STAI) | Anxiety | Breast Cancer | mindfulness-based intervention (MBI) |
| Hsiao et al. 2016 | 18–65 years | Female: 100% of survivors | 0.075 | Beck Depression Inventory (BDI-II) | Depression | Breast Cancer | mindfulness-based intervention (MBI) |
| Johannsen et al. 2016^21^ | Mean not directly reported; participants were women aged ≥3 months post-treatment (n=129) | Female: 100% (N = 129; MBCT n=67, Control n=62) | 20–30% | HADS (Hospital Anxiety and Depression) | Depression and Anxiety | Breast Cancer | Mindfulness-based cognitive therapy (MBCT) |
| Johns et al. 2014^22^ | MBSR: 58.8 ± 9.3 yrs; Control: 55.7 ± 9.3 yrs | Female: 94% (N=33), Male: 6% (N=2); N=35 total | 0.03 | GAD-7 (Anxiety) | Anxiety | cancer | MindfulnessBased Stress Reduction (MBSR) |
| Johns et al. 2014 | MBSR: 58.8 ± 9.3 yrs; Control: 55.7 ± 9.3 yrs | Female: 94% (N=33), Male: 6% (N=2); N=35 total | 0.03 | PHQ-8 (Depression) | Depression | cancer | MindfulnessBased Stress Reduction (MBSR) |
| Johns et al. 2016 | MBSR: 56.9 ± 9.9 yrs; PES: 56.4 ± 12.7 yrs | Female: 90.1% (N = 71; 60 breast, 11 colorectal) | 0.06 | GAD-7 (Anxiety) | Anxiety | Breast Cancer | mindfulness-based stress reduction (MBSR) |
| Johns et al. 2016^23^ | MBSR: 56.9 ± 9.9 yrs; PES: 56.4 ± 12.7 yrs | Female: 90.1% (N = 71; 60 breast, 11 colorectal) | 0.06 | PHQ-8 (Depression) | Depression | Breast Cancer | mindfulness-based stress reduction (MBSR) |
| Johns et al. 2020 | Not explicitly reported; sample = advanced cancer adults, mean likely late 50s–60s (N=13 patients, 13 caregivers) | Patients: 11 (gender not specified; majority Caucasian, English-speaking) | 0.15 | GAD-7 (Anxiety) | Anxiety | Metastatic Cancer | mindfulness-based intervention (MBI) |
| Johns et al. 2020^24^ | Not explicitly reported; sample = advanced cancer adults, mean likely late 50s–60s (N=13 patients, 13 caregivers) | Patients: 11 (gender not specified; majority Caucasian, English-speaking) | 0.15 | PHQ-8 (Depression) | Depression | Metastatic Cancer | mindfulness-based intervention (MBI) |
| Juarez-Reyes et al. 2024^25^ | 51.5 ± 8.0 years | Female: 100% (N = 31; 16 intervention, 14 control after 1 withdrew) | ≥ 94% | GAD-7 (Anxiety) | Anxiety | Breast Cancer | mindfulness-based intervention (MBI) |
| Juarez-Reyes et al. 2024 | 51.5 ± 8.0 years | Female: 100% (N = 31; 16 intervention, 14 control after 1 withdrew) | ≥ 94% | CES-D (Depression) | Depression | Breast Cancer | mindfulness-based intervention (MBI) |
| Sarenmalm et al. 2017^26^ | 57.2 ± 10.2 years (range 34–80) | Female: 100% (N = 166 randomized; MBSR n=62, Active Control n=52, Non-MBSR n=52) | 11 | HADS (Hospital Anxiety and Depression Scale) | Anxiety | Breast Cancer | mindfulness-based stress reduction (MBSR) |
| Sarenmalm et al. 2017 | 57.2 ± 10.2 years (range 34–80) | Female: 100% (N = 166 randomized; MBSR n=62, Active Control n=52, Non-MBSR n=52) | 11 | HADS (Hospital Anxiety and Depression Scale) | Depression | Breast Cancer | mindfulness-based stress reduction (MBSR) |
| Lengacher et a 2012^27^ | Not explicitly reported; patients aged ≥21 yrs; advanced-stage breast, colon, lung, prostate | Mixed, ~31% breast cancer; N=26 patients (gender breakdown not reported, but includes men & women) | 0.029 | PSS (Perceived Stress) | Stress | Cancer | mindfulness-based stress reduction (MBSR) |
| Lengacher et a 2012 | Not explicitly reported; patients aged ≥21 yrs; advanced-stage breast, colon, lung, prostate | Mixed, ~31% breast cancer; N=26 patients (gender breakdown not reported, but includes men & women) | 0.029 | Trait Anxiety | Anxiety | Cancer | mindfulness-based stress reduction (MBSR) |
| Lengacher et a 2012 | Not explicitly reported; patients aged ≥21 yrs; advanced-stage breast, colon, lung, prostate | Mixed, ~31% breast cancer; N=26 patients (gender breakdown not reported, but includes men & women) | 0.029 | State Anxiety | Anxiety | Cancer | mindfulness-based stress reduction (MBSR) |
| Lengacher et a 2012 | Not explicitly reported; patients aged ≥21 yrs; advanced-stage breast, colon, lung, prostate | Mixed, ~31% breast cancer; N=26 patients (gender breakdown not reported, but includes men & women) | 0.029 | CES-D (Depression) | Depression | Cancer | mindfulness-based stress reduction (MBSR) |
| Lengacher et a 2016^28^ | 56.6 ± 9.7 yrs (range 21+, stages 0–III, 2 wks–2 yrs post-treatment) | Female: 100% (N = 322; MBSR=167, UC=155) | 0.07 | State-Trait Anxiety Inventory–State | Anxiety | Breast Cancer | mindfulness-based stress reduction (MBSR) |
| Lengacher et a 2016 | 56.6 ± 9.7 yrs (range 21+, stages 0–III, 2 wks–2 yrs post-treatment) | Female: 100% (N = 322; MBSR=167, UC=155) | 0.07 | CES-D (Depression) | Depression | Breast Cancer | mindfulness-based stress reduction (MBSR) |
| Matousek et al. 2011^29^ | 55.9 ± 10.8 yrs (range 28–72) | Female: 100% (N = 33; all breast cancer survivors) | N/A | PSS-10 (Perceived Stress) | Depression | Breast Cancer | Mindfulness-Based Stress Reduction (MBSR) |
| Matousek et al. 2011 | 55.9 ± 10.8 yrs (range 28–72) | Female: 100% (N = 33; all breast cancer survivors) | N/A | CES-D (Depression) | Stress | Breast Cancer | Mindfulness-Based Stress Reduction (MBSR) |
| Messer et al. 2019^30^ | 51.0 yrs (mean; SD not reported) | Female: 76%, Male: 24% (N=23; Stage I–III survivors, mixed cancer types) | 0.029 | HADS (Hospital Anxiety & Depression Scale) | Depression and Anxiety | Cancer | mindfulness-based intervention (MBI) |
| Zhu et al . 2023 | 48.88 ± 8.02 yrs | Female: 100% (N = 101; MBSR=50, Control=51) | 0.029 | SAS (Self-Rating Anxiety Scale) | Anxiety | Breast Cancer | Mindfulness-Based Stress Reduction (MBSR) |
| Zhu et al . 2023^31^ | 48.88 ± 8.02 yrs | Female: 100% (N = 101; MBSR=50, Control=51) | 0.029 | SDS (Self-Rating Depression Scale) | Depression | Breast Cancer | Mindfulness-Based Stress Reduction (MBSR) |
| Mirmahmoodi et al . 2020^32^ | Control: 45.64 ± 10.11 yrs; Intervention: 44.14 ± 11.19 yrs | Female: 100% (N = 44; 22 intervention, 22 control) | N/A | Perceived Stress Scale (PSS) | Stress | Breast Cancer | Mindfulness-Based Stress Reduction (MBSR) |
| Mirmahmoodi et al . 2020 | Control: 45.64 ± 10.11 yrs; Intervention: 44.14 ± 11.19 yrs | Female: 100% (N = 44; 22 intervention, 22 control) | N/A | Beck Anxiety Inventory (BAI) | Anxiety | Breast Cancer | Mindfulness-Based Stress Reduction (MBSR) |
| Mirmahmoodi et al . 2020 | Control: 45.64 ± 10.11 yrs; Intervention: 44.14 ± 11.19 yrs | Female: 100% (N = 44; 22 intervention, 22 control) | N/A | Beck Depression Inventory-II (BDI-II) | Depression | Breast Cancer | Mindfulness-Based Stress Reduction (MBSR) |
| Nissim et al . 2019 | 34 ± 6.3 yrs (range 19–45) | Female: 86% (N=70; 60 women, 10 men) | 0.08 | PSS-10 (Perceived Stress) | Stress | Cancer | Mindfulness-based cognitive therapy (MBCT) |
| Nissim et al . 2019^33^ | 35 ± 6.3 yrs (range 19–45) | Female: 86% (N=70; 60 women, 10 men) | 0.08 | GAD-7 (Anxiety) | Anxiety | Cancer | Mindfulness-based cognitive therapy (MBCT) |
| Nissim et al. 2019 | 36 ± 6.3 yrs (range 19–45) | Female: 86% (N=70; 60 women, 10 men) | 0.08 | PHQ-9 (Depression) | Depression | Cancer | Mindfulness-based cognitive therapy (MBCT) |
| Park et al . 2017^34^ | 50.1 ± 9.1 yrs (range 20–75) | Female: 100% (N = 12; Stage I–II breast cancer) | N/A | HADS (Hospital Anxiety & Depression Scale – anxiety, depression) | Anxiety | Breast Cancer | Mindfulness-based cognitive therapy (MBCT) |
| Park et al . 2017 | 50.1 ± 9.1 yrs (range 20–75) | Female: 100% (N = 12; Stage I–II breast cancer) | N/A | HADS (Hospital Anxiety & Depression Scale – anxiety, depression) | Depression | Breast Cancer | Mindfulness-based cognitive therapy (MBCT) |
| Pollard et al. 2016^35^ | Majority ≥ 60 yrs (58%); range 20–70+ yrs; mean not reported | Male: 68% (n=13), Female: 32% (n=6); total N=19 | 0.029 | POMS-SF (Profile of Mood States – anxiety) | Depression | Neck Cancer | Mindfulness-Based Stress Reduction (MBSR) |
| Cohen et al. 2022^36^ | 59.05 ± 11.34 yrs (range 31–73) | Female: 100% (N=33; ovarian cancer survivors) | 0.3 | HADS (Anxiety, Depression) | Anxiety | Ovarian Cancer | mindfulness-based cognitive therapy (MBCT) |
| Cohen et al. 2022 | 59.05 ± 11.34 yrs (range 31–73) | Female: 100% (N=33; ovarian cancer survivors) | 0.3 | HADS (Anxiety, Depression) | Depression | Ovarian Cancer | mindfulness-based cognitive therapy (MBCT) |
| Stafford et al. 2013^37^ | 50.15 ± 10.0 yrs (range 31–66) | Female: 100% (N=42 completers; 71% breast, 29% gynecologic cancers) | 0.16 | DASS-21 (Depression, Anxiety, Stress Scale), FACT-G (Quality of Life), Freiburg Mindfulness Inventory (FMI), PTGI (Post-Traumatic Growth) | Depression , Anxiety and Stress | Breast Cancer | Mindfulness-Based Cognitive Therapy (MBCT) |
| Thornton et al. 2014^38^ | 58 yrs (mean; SD not reported) | Female: 100% (N = 32; breast = 18 [56%], gynecologic = 14 [44%]) | N/A | Anxiety (GAD symptoms, Worry) | Anxiety | Cancer | mindfulness-based intervention (MBI) |
| Thornton et al. 2014 | 59 yrs (mean; SD not reported) | Female: 100% (N = 32; breast = 18 [56%], gynecologic = 14 [44%]) | N/A | Depression (BDI-II) | Depression | Cancer | mindfulness-based intervention (MBI) |
| Victorson et al. 2020^39^ | 32.8 ± 4.76 yrs (range 18–39, YA sample; avg 2 yrs since dx) | Female: 79% (N=126 randomized; 67 MBSR, 59 waitlist; breast cancer = 34%) | N/A | PROMIS-CATs (Anxiety, Depression) | Anxiety | Cancer | mindfulness-based stress reduction (MBSR) |
| Victorson et al. 2020 | 32.8 ± 4.76 yrs (range 18–39, YA sample; avg 2 yrs since dx) | Female: 79% (N=126 randomized; 67 MBSR, 59 waitlist; breast cancer = 34%) | N/A | PROMIS-CATs (Anxiety, Depressione) | Depression | Cancer | mindfulness-based stress reduction (MBSR) |
| Janusek, et al. 2019^40^ | MBSR: 55.0 ± 10.1 yrs; ACC: 55.2 ± 10.1 yrs | Female: 100% (N=164 randomized; 84 MBSR, 80 ACC; all breast cancer, Stage 0–III) | N/A | PSS (Perceived Stress) | Stress | Cancer | mindfulness-based stress reduction (MBSR) |
| Janusek, et al. 2019 | MBSR: 55.0 ± 10.1 yrs; ACC: 55.2 ± 10.1 yrs | Female: 100% (N=164 randomized; 84 MBSR, 80 ACC; all breast cancer, Stage 0–III) | N/A | CES-D (Depression) | Depression | Cancer | mindfulness-based stress reduction (MBSR) |
| Zernicke et al. 2014^41^ | 58 ± 8.2 yrs (MBCR), 58 ± 13.0 yrs (WL) | Female: 73% (n=45/62), Male: 27% (n=17/62); Breast cancer: 34% (n=21) | 0.167 | POMS (Profile of Mood States – anxiety, depression, mood disturbance) | Stress | Cancer | mindfulness basedcognitivetherapy(MBCT) |
| Zhang et al. 2016^42^ | 46.10 ± 6.43 yrs (range 30–62) | Female: 100% (N=60; Stages I–III BC) | 0.033 | Chinese Perceived Stress Scale | Stress | Breast Cancer | mindfulness-based stress reduction (MBSR) |
| Zhang et al. 2016 | 46.10 ± 6.43 yrs (range 30–62) | Female: 100% (N=60; Stages I–III BC) | 0.033 | STAI (State Anxiety Inventory) | Anxiety | Breast Cancer | mindfulness-based stress reduction (MBSR) |
| Zhang et al. 2016 | 46.10 ± 6.43 yrs (range 30–62) | Female: 100% (N=60; Stages I–III BC) | 0.033 | STAI (Trait Anxiety Inventory) | Anxiety | Breast Cancer | mindfulness-based stress reduction (MBSR) |
| Ruixing Zhang et al. 2017 | Exp: 38.35 ± 8.93 yrs; Ctrl: 39.71 ± 9.42 yrs (range 17–71) | Male: 19 (58%) / Female: 14 (42%) Exp; Male: 17 (53%) / Female: 15 (47%) Ctrl; N=70 (33 Exp, 32 Ctrl completers) | 0.066 | SAS (Self-Rating Anxiety Scale) | Anxiety | Leukemia | mindfulness-based intervention (MBI) |
| Ruixing Zhang et al. 2017^43^ | Exp: 38.35 ± 8.93 yrs; Ctrl: 39.71 ± 9.42 yrs (range 17–71) | Male: 19 (58%) / Female: 14 (42%) Exp; Male: 17 (53%) / Female: 15 (47%) Ctrl; N=70 (33 Exp, 32 Ctrl completers) | 0.066 | SDS (Self-Rating Depression Scale | Depression | Leukemia | mindfulness-based intervention (MBI) |

**eTable 3. Effects of MBIs on Depression, Anxiety, and Stress by Cancer Type**

| Outcome | Cancer Type | No. of Studies (k) | SMD (95% CI) | I² (%) | Between-group p-value |
| --- | --- | --- | --- | --- | --- |
| Depression |  | 34 | –0.92 [–1.31, –0.53] | 94.1 | — |
|  | Breast Cancer | 16 | –1.50 [–2.21, –0.78] | 95.5 |  |
|  | Genitourinary Cancer | 1 | –0.12 [–0.93, 0.68] | — |  |
|  | Gynaecologic Cancer | 2 | –0.23 [–1.10, 0.64] | 0 |  |
|  | Hematologic Cancer | 1 | –1.60 [–2.16, –1.04] | — |  |
|  | Mixed Cancer Survivors | 3 | –0.52 [–0.70, –0.33] | 0 |  |
|  | Other Cancer Types | 11 | –0.31 [–0.65, 0.04] | 88.2 |  |
|  | Subgroup p-value |  |  |  | < 0.0001 |
| Anxiety |  | 32 | –1.06 [–1.67, –0.46] | 93.9 | — |
|  | Breast Cancer | 15 | –1.44 [–2.63, –0.25] | 96.4 |  |
|  | Genitourinary Cancer | 2 | –0.07 [–1.23, 1.09] | 0 |  |
|  | Gynaecologic Cancer | 2 | –0.35 [–1.98, 1.28] | 0 |  |
|  | Hematologic Cancer | 1 | –1.92 [–2.51, –1.33] | — |  |
|  | Mixed Cancer Survivors | 2 | –0.57 [–1.14, –0.00] | 0 |  |
|  | Other Cancer Types | 10 | –0.85 [–1.87, 0.17] | 88.1 |  |
|  | Subgroup p-value |  |  |  | < 0.0001 |
| Stress |  | 14 | –1.50 [–2.48, –0.51] | 95.5 | — |
|  | Breast Cancer | 7 | –1.86 [–3.60, –0.13] | 96.5 |  |
|  | Mixed Cancer Survivors | 2 | –0.64 [–3.84, 2.55] | 30.1 |  |
|  | Other Cancer Types | 5 | –1.36 [–3.69, 0.97] | 94.6 |  |
|  | Subgroup p-value |  |  |  | 0.2159 |

Abbreviations: *g*, Hedges’ g; CI, confidence interval; MBI, mindfulness-based intervention; MBSR, Mindfulness-Based Stress Reduction; MBCT, Mindfulness-Based Cognitive Therapy.

**eTable 4**. Meta-Regression of Effect Sizes by Mental Health Outcome

| Model | Estimate (b) | SE | 95% CI | t/ z | p-value |
| --- | --- | --- | --- | --- | --- |
| Main RVE Model | –0.88 | 0.17 | [–1.21, –0.54] | –5.28 | < .001 *** |
| Subgroup: Outcome Type |  |  |  |  |  |
| Intercept (Anxiety) | –0.93 | 0.22 | [–1.36, –0.49] | –4.29 | < .001 *** |
| Both Conditions vs Anxiety | 0.53 | 0.26 | [0.01, 1.06] | 2.03 | .048 * |
| Depression vs Anxiety | 0.15 | 0.1 | [–0.04, 0.35] | 1.56 | 0.126 |
| Stress vs Anxiety | –0.36 | 0.25 | [–0.86, 0.15] | –1.43 | 0.16 |

**Note: Model Fit & Heterogeneity**

- **Main RVE I²**: 93.3%
- **Tau²** (between-study variance): 1.30
- **Moderator Test** (*F*(3, 45) = 3.41): *p* = .025
- Traditional random-effects estimate (no RVE): **g = –1.03**, 95% CI [–1.33, –0.74], I² = 97.1%

eR Code using for the analysis

# ===============================

# Subgroup meta-analysis by Mental Health (Oncology2.xlsx)

# Hedges' g, random-effects (REML), Hartung–Knapp CIs

# ===============================

if (!requireNamespace("meta", quietly = TRUE)) install.packages("meta")

if (!requireNamespace("readxl", quietly = TRUE)) install.packages("readxl")

library(meta)

library(readxl)

# --- Load

Oncology2 <- read_excel("C:/Users/*************************************************************")

# --- Clean column names: spaces/dashes -> "_", drop parentheses

names(Oncology2) <- gsub("[[:space:]-]+", "_", names(Oncology2))

names(Oncology2) <- gsub("[()]", "", names(Oncology2))

# --- Standardize key columns

# Study

stud_col <- grep("^study", names(Oncology2), ignore.case = TRUE, value = TRUE)

stopifnot(length(stud_col) >= 1)

names(Oncology2)[names(Oncology2) == stud_col[1]] <- "Study"

# Mental health

mh_col <- grep("^Mental_health", names(Oncology2), ignore.case = TRUE, value = TRUE)

stopifnot(length(mh_col) >= 1)

names(Oncology2)[names(Oncology2) == mh_col[1]] <- "Mental_Health"

# Coerce numeric columns for metacont

num_cols <- c("Control_mean","Control_sd","Control_n",

"Intervention_mean","Intervention_sd","Intervention_n")

missing <- setdiff(num_cols, names(Oncology2))

if (length(missing)) stop(sprintf("Missing required columns: %s", paste(missing, collapse=", ")))

for (cc in num_cols) Oncology2[[cc]] <- suppressWarnings(as.numeric(as.character(Oncology2[[cc]])))

# --- Normalize Mental_Health values

mh <- tolower(trimws(Oncology2$Mental_Health))

both_mask <- grepl("both", mh) | (grepl("anx", mh) & grepl("dep", mh))

mh_clean <- ifelse(both_mask, "Both Conditions",

ifelse(grepl("^dep|depress", mh), "Depression",

ifelse(grepl("^anx", mh), "Anxiety",

ifelse(grepl("^stress", mh), "Stress", NA))))

Oncology2$Mental_Health <- factor(mh_clean,

levels = c("Depression","Anxiety","Stress","Both Conditions"))

# --- Prepare analysis data

dat <- Oncology2[complete.cases(Oncology2[, num_cols]) & !is.na(Oncology2$Mental_Health),

c(num_cols, "Study", "Mental_Health")]

if (nrow(dat) < 2) stop("Not enough studies after cleaning to run a meta-analysis.")

# --- Subgroup meta by Mental Health

m_by_mh <- metacont(

n.e = dat$Intervention_n,

mean.e = dat$Intervention_mean,

sd.e = dat$Intervention_sd,

n.c = dat$Control_n,

mean.c = dat$Control_mean,

sd.c = dat$Control_sd,

studlab = dat$Study,

data = dat,

sm = "SMD", # Hedges' g

method.smd = "Hedges",

common = FALSE,

random = TRUE,

method.tau = "REML",

method.random.ci = "HK",

byvar = dat$Mental_Health # <-- subgrouping here

)

# Outputs

print(table(dat$Mental_Health))

print(summary(m_by_mh), digits = 2)

# --- Forest plot

forest(m_by_mh,

xlab = "Standardized Mean Difference (Hedges' g)",

print.byvar = TRUE,

print.Q = TRUE,

print.I2 = TRUE,

print.tau2 = TRUE,

overall = TRUE,

overall.hetstat = TRUE,

main = "Oncology2 — Subgroup Meta-analysis by Mental Health")

# # OPTIONAL: Save to PDF

# pdf("oncology2_by_mental_health_forest.pdf", width = 8.5, height = 11)

# forest(m_by_mh,

# xlab = "Standardized Mean Difference (Hedges' g)",

# print.byvar = TRUE, print.Q = TRUE, print.I2 = TRUE, print.tau2 = TRUE,

# overall = TRUE, overall.hetstat = TRUE,

# main = "Oncology2 — Subgroup Meta-analysis by Mental Health")

# dev.off()

# ===============================

# Oncology2: overall + by Mental Health

# Meta (Hedges' g), funnel, Egger (k>=10), trim-and-fill

# ===============================

if (!requireNamespace("meta", quietly = TRUE)) install.packages("meta")

if (!requireNamespace("readxl", quietly = TRUE)) install.packages("readxl")

library(meta)

library(readxl)

# --- Load

Oncology2 <- read_excel("C:/Users/**************")

# --- Clean column names

names(Oncology2) <- gsub("[[:space:]-]+", "_", names(Oncology2))

names(Oncology2) <- gsub("[()]", "", names(Oncology2))

# Standardize key columns

stud_col <- grep("^study", names(Oncology2), ignore.case = TRUE, value = TRUE)[1]

if (is.na(stud_col)) stop("Couldn't find Study column")

names(Oncology2)[names(Oncology2) == stud_col] <- "Study"

mh_col <- grep("^Mental_health", names(Oncology2), ignore.case = TRUE, value = TRUE)[1]

if (is.na(mh_col)) stop("Couldn't find Mental health column")

names(Oncology2)[names(Oncology2) == mh_col] <- "Mental_Health"

# Coerce numeric columns for metacont

num_cols <- c("Control_mean","Control_sd","Control_n",

"Intervention_mean","Intervention_sd","Intervention_n")

miss <- setdiff(num_cols, names(Oncology2))

if (length(miss)) stop(sprintf("Missing columns: %s", paste(miss, collapse=", ")))

for (cc in num_cols) Oncology2[[cc]] <- suppressWarnings(as.numeric(as.character(Oncology2[[cc]])))

# Normalize Mental_Health values

mh <- tolower(trimws(Oncology2$Mental_Health))

both_mask <- grepl("both", mh) | (grepl("anx", mh) & grepl("dep", mh))

mh_clean <- ifelse(both_mask, "Both Conditions",

ifelse(grepl("^dep|depress", mh), "Depression",

ifelse(grepl("^anx", mh), "Anxiety",

ifelse(grepl("^stress", mh), "Stress", NA))))

Oncology2$Mental_Health <- factor(mh_clean,

levels = c("Depression","Anxiety","Stress","Both Conditions"))

# Keep complete numeric rows

dat <- Oncology2[complete.cases(Oncology2[, num_cols]), c(num_cols, "Study", "Mental_Health")]

if (nrow(dat) < 2) stop("Not enough complete rows to run meta-analysis.")

# --- Overall meta-analysis (random-effects, Hedges' g, REML, HK)

m_all <- metacont(

n.e = dat$Intervention_n,

mean.e = dat$Intervention_mean,

sd.e = dat$Intervention_sd,

n.c = dat$Control_n,

mean.c = dat$Control_mean,

sd.c = dat$Control_sd,

studlab = dat$Study,

data = dat,

sm = "SMD", method.smd = "Hedges",

common = FALSE, random = TRUE,

method.tau = "REML",

method.random.ci = "HK"

)

print(summary(m_all), digits = 2)

# --- Subgroup meta by Mental Health

if (sum(!is.na(dat$Mental_Health)) >= 2) {

m_by_mh <- metacont(

n.e = dat$Intervention_n,

mean.e = dat$Intervention_mean,

sd.e = dat$Intervention_sd,

n.c = dat$Control_n,

mean.c = dat$Control_mean,

sd.c = dat$Control_sd,

studlab = dat$Study,

data = dat,

sm = "SMD", method.smd = "Hedges",

common = FALSE, random = TRUE,

method.tau = "REML",

method.random.ci = "HK",

byvar = dat$Mental_Health

)

print(summary(m_by_mh), digits = 2)

forest(m_by_mh,

xlab = "Standardized Mean Difference (Hedges' g)",

print.byvar = TRUE, print.Q = TRUE, print.I2 = TRUE, print.tau2 = TRUE,

overall = TRUE, overall.hetstat = TRUE,

main = "Oncology2 — Subgrouped by Mental Health")

}

# ===============================

# Funnels + Egger (k≥10) + trim-and-fill (meta::trimfill)

# ===============================

run_bias_checks <- function(meta_obj,

label = "Overall",

min_k_bias = 10,

save_prefix = NULL,

ask = FALSE) {

k_used <- sum(is.finite(meta_obj$TE))

cat(sprintf("\n[%s] Studies included: k = %d\n", label, k_used))

old_ask <- par("ask"); on.exit(par(ask = old_ask), add = TRUE)

par(ask = isTRUE(ask))

# 1) Original funnel (always)

if (is.null(save_prefix)) {

funnel(meta_obj,

xlab = "Effect size (SMD, Hedges' g)",

ylab = "Standard Error",

main = sprintf("Funnel — %s (original)", label))

} else {

pdf(paste0(save_prefix, "_funnel_original.pdf"), width = 7, height = 9)

funnel(meta_obj,

xlab = "Effect size (SMD, Hedges' g)",

ylab = "Standard Error",

main = sprintf("Funnel — %s (original)", label))

dev.off()

}

# 2) Only if k ≥ threshold: Egger + trim-and-fill

if (k_used < min_k_bias) {

cat(sprintf("[%s] k < %d: Skipping Egger and trim-and-fill.\n", label, min_k_bias))

return(invisible(NULL))

}

eg <- metabias(meta_obj, method.bias = "linreg")

cat(sprintf("[%s] Egger’s test:\n", label)); print(eg)

tf <- trimfill(meta_obj) # meta::trimfill

cat(sprintf("[%s] Trim-and-fill summary:\n", label)); print(summary(tf))

# 3) Funnel after trim-and-fill

if (is.null(save_prefix)) {

funnel(tf,

xlab = "Effect size (SMD, Hedges' g) — trim-and-fill",

ylab = "Standard Error",

main = sprintf("Funnel — %s (trim-and-fill)", label))

} else {

pdf(paste0(save_prefix, "_funnel_trimfill.pdf"), width = 7, height = 9)

funnel(tf,

xlab = "Effect size (SMD, Hedges' g) — trim-and-fill",

ylab = "Standard Error",

main = sprintf("Funnel — %s (trim-and-fill)", label))

dev.off()

}

invisible(list(egger = eg, trimfill = tf))

}

# --- Run funnels/bias checks (Plots pane in RStudio)

run_bias_checks(m_all, label = "Overall", min_k_bias = 10)

# Optional: per mental health category

if (exists("m_by_mh")) {

for (lev in levels(na.omit(dat$Mental_Health))) {

idx <- which(dat$Mental_Health == lev)

if (length(idx) >= 2) {

m_sub <- update.meta(m_all, subset = dat$Mental_Health == lev)

run_bias_checks(m_sub, label = paste("Mental Health:", lev), min_k_bias = 10)

}

}

}

# ===============================

# ONE forest plot with subgroups:

# Adapted/Modified MBIs by Mental Health (Depression, Anxiety, Stress, Both)

# ===============================

# Packages

if (!requireNamespace("meta", quietly = TRUE)) install.packages("meta")

if (!requireNamespace("readxl", quietly = TRUE)) install.packages("readxl")

library(meta)

library(readxl)

# Load

Oncology1 <- read_excel("C:/Users/*******************************")

# --- Robust column cleaning ---

# Replace spaces/dashes with underscores and drop parentheses

names(Oncology1) <- gsub("[[:space:]-]+", "_", names(Oncology1))

names(Oncology1) <- gsub("[()]", "", names(Oncology1))

# Standardize key column names

# study -> Study

stud_col <- grep("^study", names(Oncology1), ignore.case = TRUE, value = TRUE)

if (length(stud_col) >= 1) names(Oncology1)[names(Oncology1) == stud_col[1]] <- "Study"

# Mental health column -> Mental_Health

mh_col <- grep("^Mental_health", names(Oncology1), ignore.case = TRUE, value = TRUE)

if (length(mh_col) == 0) stop("Could not find the mental health column.")

names(Oncology1)[names(Oncology1) == mh_col[1]] <- "Mental_Health"

# MBIs column -> MBI_Type

mbi_col <- grep("^MBIs$", names(Oncology1), ignore.case = TRUE, value = TRUE)

if (length(mbi_col) == 0) stop("Could not find the MBIs column.")

names(Oncology1)[names(Oncology1) == mbi_col[1]] <- "MBI_Type"

# Numeric columns (means/SDs/ns)

num_cols <- c("Control_mean","Control_sd","Control_n",

"Intervention_mean","Intervention_sd","Intervention_n")

# Safely coerce numerics

for (cc in num_cols) {

if (cc %in% names(Oncology1)) {

Oncology1[[cc]] <- suppressWarnings(as.numeric(as.character(Oncology1[[cc]])))

} else {

stop(sprintf("Missing column '%s' in the sheet.", cc))

}

}

# --- Normalize Mental_Health values ---

mh <- tolower(trimws(Oncology1$Mental_Health))

both_mask <- grepl("both", mh) | (grepl("anx", mh) & grepl("dep", mh)) # any text containing both

mh_clean <- ifelse(both_mask, "Both Conditions",

ifelse(grepl("^dep|depress", mh), "Depression",

ifelse(grepl("^anx|anxie|anxiety", mh), "Anxiety",

ifelse(grepl("^stress", mh), "Stress", NA))))

Oncology1$Mental_Health <- factor(mh_clean,

levels = c("Depression","Anxiety","Stress","Both Conditions"))

# --- Normalize MBI_Type values; map typos/variants ---

mbi <- tolower(trimws(Oncology1$MBI_Type))

mbi <- gsub("mbs\\)?$", "mbsr", mbi) # fix "MBS)" -> "mbsr"

mbi_norm <- ifelse(grepl("adapted|modified", mbi), "Adapted or Modified MBIs",

ifelse(grepl("mbsr", mbi), "MBSR",

ifelse(grepl("mbct", mbi), "MBCT",

ifelse(grepl("mbcr", mbi), "MBCR",

ifelse(grepl("\\bmbi\\b", mbi), "MBI (unspecified)", NA)))))

Oncology1$MBI_Type <- factor(mbi_norm,

levels = c("MBSR","MBCT","MBCR","Adapted or Modified MBIs","MBI (unspecified)"))

# --- Keep only Adapted/Modified MBIs and complete rows ---

dat <- subset(Oncology1, MBI_Type == "Adapted or Modified MBIs" & !is.na(Mental_Health))

dat <- na.omit(dat[, c("Intervention_n","Intervention_mean","Intervention_sd",

"Control_n","Control_mean","Control_sd",

"Study","Mental_Health","MBI_Type")])

if (nrow(dat) < 2) stop("Not enough Adapted/Modified MBI studies after cleaning to run a meta-analysis.")

# (Optional) Quick counts per subgroup

print(table(dat$Mental_Health))

# --- Meta-analysis with subgroups = Mental_Health ---

m <- metacont(

n.e = dat$Intervention_n,

mean.e = dat$Intervention_mean,

sd.e = dat$Intervention_sd,

n.c = dat$Control_n,

mean.c = dat$Control_mean,

sd.c = dat$Control_sd,

studlab = dat$Study,

data = dat,

sm = "SMD", # Hedges' g

method.smd = "Hedges",

common = FALSE,

random = TRUE,

method.tau = "REML",

method.random.ci = "HK",

byvar = dat$Mental_Health # subgrouping by mental health condition

)

print(summary(m), digits = 2)

# ---- Forest plot ----

forest(m,

xlab = "Standardized Mean Difference (Hedges' g)",

print.byvar = TRUE,

print.Q = TRUE,

print.I2 = TRUE,

print.tau2 = TRUE,

overall = TRUE,

overall.hetstat = TRUE,

main = "Adapted/Modified MBIs in Oncology (Subgrouped by Mental Health Condition)")

# OPTIONAL: Save to PDF

# pdf("adapted_modified_by_mental_health.pdf", width = 8.5, height = 11)

# forest(m,

# xlab = "Standardized Mean Difference (Hedges' g)",

# print.byvar = TRUE, print.Q = TRUE, print.I2 = TRUE, print.tau2 = TRUE,

# overall = TRUE, overall.hetstat = TRUE,

# main = "Adapted/Modified MBIs in Oncology (Subgrouped by Mental Health Condition)")

# dev.off()

eList of included studies References

1. Park S, Sato Y, Takita Y, et al. Mindfulness-Based Cognitive Therapy for Psychological Distress, Fear of Cancer Recurrence, Fatigue, Spiritual Well-Being, and Quality of Life in Patients With Breast Cancer—A Randomized Controlled Trial. *J Pain Symptom Manage*. 2020;60(2):381-389. doi:10.1016/j.jpainsymman.2020.02.017

2. Russell L, Ugalde A, Orellana L, et al. A pilot randomised controlled trial of an online mindfulness-based program for people diagnosed with melanoma. *Support Care Cancer*. 2019;27(7):2735-2746. doi:10.1007/s00520-018-4574-6

3. Bränström R, Kvillemo P, Brandberg Y, Moskowitz JT. Self-report mindfulness as a mediator of psychological well-being in a stress reduction intervention for cancer patients—A randomized study. *Ann Behav Med*. 2010;39(2):151-161. doi:10.1007/s12160-010-9168-6

4. Price-Blackshear M, Pratscher S, Oyler D, et al. Online couples mindfulness-based intervention for young breast cancer survivors and their partners: A randomized-control trial. *J Psychosoc Oncol*. 2020;38(5):592-611. doi:10.1080/07347332.2020.1778150

5. Arden-Close E, Mitchell F, Davies G, et al. Mindfulness-based interventions in recurrent ovarian cancer: A mixed-methods feasibility study. *Integr Cancer Ther*. 2020;19:1534735420908341. doi:10.1177/1534735420908341

6. Boyle CC, Stanton AL, Ganz PA, Crespi CM, Bower JE. Improvements in emotion regulation following mindfulness meditation: Effects on depressive symptoms and perceived stress in younger breast cancer survivors. *J Consult Clin Psychol*. 2017;85(4):397-402. doi:10.1037/ccp0000186

7. Bruggeman Everts FZ, van der Lee ML, de Jager Meezenbroek E. Web-based individual Mindfulness-Based Cognitive Therapy for cancer-related fatigue — A pilot study. *Internet Interv*. 2015;2(2):200-213. doi:10.1016/j.invent.2015.03.004

8. Bower J, Crosswell A, Stanton A, et al. Mindfulness Meditation for Younger Breast Cancer Survivors: A Randomized Controlled Trial. *CANCER*. 2015;121(8):1231-1240. doi:10.1002/cncr.29194

9. Cavanagh K, Strauss C, Cicconi F, Griffiths N, Wyper A, Jones F. A randomised controlled trial of a brief online mindfulness-based intervention. *Behav Res Ther*. 2013;51(9):573-578. doi:10.1016/j.brat.2013.06.003

10. Chambers SK, Occhipinti S, Foley E, et al. Mindfulness-based cognitive therapy in advanced prostate cancer: A randomized controlled trial. *J Clin Oncol*. 2016;35(3):291-297. doi:10.1200/JCO.2016.68.8788

11. Cheli S, Caligiani L, Martella F, De Bartolo P, Mancini F, Fioretto L. Mindfulness and metacognition in facing with fear of recurrence: A proof‐of‐concept study with breast‐cancer women. *Psychooncology*. 2019;28(3):600-606. doi:10.1002/pon.4984

12. Wang YL, Zhang XF, Wang XP, Zhang YJ, Jin YY, Li WL. Combined mindfulness-based stress reduction and exercise intervention for improving psychological well-being in patients with non-small cell lung cancer. *Clin Psychol Psychother*. 2024;31(4):e3023. doi:10.1002/cpp.3023

13. Dobos G, Overhamm T, Büssing A, et al. Integrating mindfulness in supportive cancer care: a cohort study on a mindfulness-based day care clinic for cancer survivors. *Support CARE CANCER*. 2015;23(10):2945-2955. doi:10.1007/s00520-015-2660-6

14. Dragomanovich HM, Dhruva A, Ekman E, et al. Being Present 2.0: Online mindfulness-based program for metastatic gastrointestinal cancer patients and caregivers. *Glob Adv Health Med*. 2021;10:21649561211044693. doi:10.1177/21649561211044693

15. Naskar S, Dixit S, Varadharasu S, Pattnaik J, Singh R. Effect of mindfulness-based intervention on perceived stress among breast cancer patients undergoing chemotherapy. *J Fam Med Prim CARE*. 2024;13(8):2934-2940. doi:10.4103/jfmpc.jfmpc_1713_23

16. Xu F, Zhang J, Xie S, Li Q. Effects of Mindfulness-Based Cancer Recovery training on anxiety, depression, post-traumatic stress disorder, and cancer-related fatigue in breast neoplasm patients undergoing chemotherapy. *Med Baltim*. 2024;103(23):e38460. doi:10.1097/MD.0000000000038460

17. Eyles C, Leydon GM, Hoffman CJ, et al. Mindfulness for the self-management of fatigue, anxiety, and depression in women with metastatic breast cancer: a mixed methods feasibility study. *Integr Cancer Ther*. 2015;14(1):42-56. doi:10.1177/1534735414546567

18. Foley E, Baillie A, Huxter M, Price M, Sinclair E. Mindfulness-Based Cognitive Therapy for Individuals Whose Lives Have Been Affected by Cancer: A Randomized Controlled Trial. *J Consult Clin Psychol*. 2010;78(1):72-79. doi:10.1037/a0017566

19. Badaghi N, Buskbjerg C, Kwakkenbos L, Bosman S, Zachariae R, Speckens A. Positive health outcomes of mindfulness-based interventions for cancer patients and survivors: A systematic review and meta-analysis. *Clin Psychol Rev*. 2024;114:102505. doi:10.1016/j.cpr.2024.102505

20. Garland EL, Manusov EG, Froeliger B, Kelly A, Williams JM, Howard MO. Mindfulness-oriented recovery enhancement for chronic pain and prescription opioid misuse: results from an early-stage randomized controlled trial. *J Consult Clin Psychol*. 2014;82(3):448-459. doi:10.1037/a0035798

21. Henderson V, Massion A, Clemow L, Hurley T, Druker S, Hébert J. A Randomized Controlled Trial of Mindfulness-Based Stress Reduction for Women With Early-Stage Breast Cancer Receiving Radiotherapy. *Integr CANCER Ther*. 2013;12(5):404-413. doi:10.1177/1534735412473640

22. Hsiao FH, Jow GM, Kuo WH, et al. The long-term effects of mindfulness added to family resilience-oriented couples support group on psychological well-being and cortisol responses in breast cancer survivors and their partners. *Mindfulness N*. 2016;7(6):1365-1376. doi:10.1007/s12671-016-0578-9

23. Johannsen M, O’Connor M, O’Toole MS, Jensen AB, Højris I, Zachariae R. Efficacy of mindfulness-based cognitive therapy on late post-treatment pain in women treated for primary breast cancer: A randomized controlled trial. *J Clin Oncol*. 2016;34(28):3390-3399. doi:10.1200/JCO.2015.65.0770

24. Johns SA, Brown LF, Beck-Coon K, Monahan PO, Tong Y, Kroenke K. Randomized controlled pilot study of mindfulness-based stress reduction for persistently fatigued cancer survivors. *Psychooncology*. 2014;24(8):885-893. doi:10.1002/pon.3648

25. Johns S, Brown L, Beck-Coon K, et al. Randomized controlled pilot trial of mindfulness-based stress reduction compared to psychoeducational support for persistently fatigued breast and colorectal cancer survivors. *Support CARE CANCER*. 2016;24(10):4085-4096. doi:10.1007/s00520-016-3220-4

26. Johns SA, Beck-Coon K, Stutz PV, et al. Mindfulness training supports quality of life and advance care planning in adults with metastatic cancer and their caregivers: Results of a pilot study. *Am J Hosp Palliat Care*. 2020;37(2):88-99. doi:10.1177/1049909119862254

27. Juarez-Reyes M, Martinez E, Xiao L, Goldman Rosas L. A Randomized Controlled Trial of a Culturally Adapted, Community-Based, Remotely Delivered Mindfulness Program for Latinx Patients With Breast Cancer is Acceptable and Feasible While Reducing Anxiety. *Glob Adv Integr Med Health*. 2024;13:27536130241274240. doi:10.1177/27536130241274240

28. Sarenmalm E, Mårtensson L, Andersson B, Karlsson P, Bergh I. Mindfulness and its efficacy for psychological and biological responses in women with breast cancer. *CANCER Med*. 2017;6(5):1108-1122. doi:10.1002/cam4.1052

29. Lengacher CA, Kip KE, Barta M, et al. A pilot study evaluating the effect of mindfulness-based stress reduction on psychological status, physical status, salivary cortisol, and interleukin-6 among advanced-stage cancer patients and their caregivers. *J Holist Nurs*. 2012;30(3):170-185. doi:10.1177/0898010111435949

30. Lengacher CA, Reich RR, Paterson CL, et al. Examination of broad symptom improvement resulting from Mindfulness-Based Stress Reduction in Breast Cancer survivors: A randomized controlled trial. *J Clin Oncol*. 2016;34(24):2827-2834. doi:10.1200/JCO.2015.65.7874

31. Matousek RH, Pruessner JC, Dobkin PL. Changes in the cortisol awakening response (CAR) following participation in mindfulness-based stress reduction in women who completed treatment for breast cancer. *Complement Ther Clin Pr*. 2011;17(2):65-70. doi:10.1016/j.ctcp.2010.10.005

32. Messer D, Horan J, Larkey L, Shanholtz C. Effects of Internet Training in Mindfulness Meditation on Variables Related to Cancer Recovery. *MINDFULNESS*. 2019;10(10):2143-2151. doi:10.1007/s12671-019-01182-y

33. Zhu P, Liu X, Shang X, Chen Y, Chen C, Wu Q. Mindfulness-based stress reduction for quality of life, psychological distress, and cognitive emotion regulation strategies in patients with Breast Cancer under early chemotherapy-a randomized controlled trial. *Holist Nurs Pr*. 2023;37(3):131-142. doi:10.1097/HNP.0000000000000580

34. Mirmahmoodi M, Mangalian P, Ahmadi A, Dehghan M. The Effect of Mindfulness-Based Stress Reduction Group Counseling on Psychological and Inflammatory Responses of the Women With Breast Cancer. *Integr CANCER Ther*. 2020;19. doi:10.1177/1534735420946819

35. Nissim RS, Roth A, Gupta AA, Elliott M. Mindfulness-based Cognitive Therapy intervention for Young Adults with cancer: A pilot mixed-method study. *J Adolesc Young Adult Oncol*. 2019;9(2):256-261. doi:10.1089/jayao.2019.0086

36. Park S, Sado M, Fujisawa D, et al. Mindfulness-based cognitive therapy for Japanese breast cancer patients—a feasibility study. *Jpn J Clin Oncol*. 2017;48(1):68-74. doi:10.1093/jjco/hyx156

37. Pollard A, Burchell JL, Castle D, et al. Individualised mindfulness-based stress reduction for head and neck cancer patients undergoing radiotherapy of curative intent: a descriptive pilot study. *Eur J Cancer Care Engl*. 2016;26(2):e12474. doi:10.1111/ecc.12474

38. Cohen P, Musiello T, Jeffares S, Bennett K. Mindfulness-based cognitive therapy for Fear of Recurrence in Ovarian Cancer Survivors (FROCS): a single-arm, open-label, pilot study. *Support CARE CANCER*. 2022;30(3):2317-2325. doi:10.1007/s00520-021-06659-y

39. Stafford L, Foley E, Judd F, Gibson P, Kiropoulos L, Couper J. Mindfulness-based cognitive group therapy for women with breast and gynecologic cancer: a pilot study to determine effectiveness and feasibility. *Support Care Cancer*. 2013;21(11):3009-3019. doi:10.1007/s00520-013-1880-x

40. Thornton LM, Cheavens JS, Heitzmann CA, Dorfman CS, Wu SM, Andersen BL. Test of mindfulness and hope components in a psychological intervention for women with cancer recurrence. *J Consult Clin Psychol*. 2014;82(6):1087-1100. doi:10.1037/a0036959

41. Victorson D, Murphy K, Benedict C, et al. A randomized pilot study of mindfulness-based stress reduction in a young adult cancer sample: Feasibility, acceptability, and changes in patient reported outcomes. *Psychooncology*. 2020;29(5):841-850. doi:10.1002/pon.5355

42. Janusek, Witek, Linda, Tell D, Mathews HL. Mindfulness based stress reduction provides psychological benefit and restores immune function of women newly diagnosed with breast cancer: A randomized trial with active control. *Brain Behav Immun*. 2019;80:358-373. doi:10.1016/j.bbi.2019.04.012

43. Zernicke K, Campbell T, Speca M, McCabe-Ruff K, Flowers S, Carlson L. A Randomized Wait-List Controlled Trial of Feasibility and Efficacy of an Online Mindfulness-Based Cancer Recovery Program: The eTherapy for Cancer Applying Mindfulness Trial. *Psychosom Med*. 2014;76(4):257-267. doi:10.1097/PSY.0000000000000053

44. Zhang JY, Zhou YQ, Feng ZW, Fan YN, Zeng GC, Wei L. Randomized controlled trial of mindfulness-based stress reduction (MBSR) on posttraumatic growth of Chinese breast cancer survivors. *Psychol Health Med*. 2016;22(1):94-109. doi:10.1080/13548506.2016.1146405

45. Zhang R, Yin J, Zhou Y. Effects of mindfulness-based psychological care on mood and sleep of leukemia patients in chemotherapy. *Int J Nurs Sci*. 2017;4(4):357-361. doi:10.1016/j.ijnss.2017.07.001
